# Bi-regional dynamic contrast-enhanced MRI for prediction of microvascular invasion in solitary BCLC stage A hepatocellular carcinoma

**DOI:** 10.1186/s13244-024-01720-w

**Published:** 2024-06-18

**Authors:** Yongjian Zhu, Bing Feng, Peng Wang, Bingzhi Wang, Wei Cai, Shuang Wang, Xuan Meng, Sicong Wang, Xinming Zhao, Xiaohong Ma

**Affiliations:** 1 Department of Diagnostic Radiology, National Cancer Center/National Clinical Research Center for Cancer/Cancer Hospital, Chinese Academy of Medical Sciences and Peking Union Medical College, Beijing, 100021 China; 2 Department of Pathology, National Cancer Center/National Clinical Research Center for Cancer/Cancer Hospital, Chinese Academy of Medical Sciences and Peking Union Medical College, Beijing, 100021 China; 3 Department of Hepatobiliary Surgery, National Cancer Center/National Clinical Research Center for Cancer/Cancer Hospital, Chinese Academy of Medical Sciences and Peking Union Medical College, Beijing, 100021 China; 4 Magnetic Resonance Imaging Research, General Electric Healthcare (China), Beijing, 100176 China

**Keywords:** Hepatocellular carcinoma, Magnetic resonance imaging, Dynamic contrast-enhanced, Microvascular invasion, Hepatectomy

## Abstract

**Objectives:**

To construct a combined model based on bi-regional quantitative dynamic contrast-enhanced magnetic resonance imaging (DCE-MRI), as well as clinical-radiological (CR) features for predicting microvascular invasion (MVI) in solitary Barcelona Clinic Liver Cancer (BCLC) stage A hepatocellular carcinoma (HCC), and to assess its ability for stratifying the risk of recurrence after hepatectomy.

**Methods:**

Patients with solitary BCLC stage A HCC were prospective collected and randomly divided into training and validation sets. DCE perfusion parameters were obtained both in intra-tumoral region (ITR) and peritumoral region (PTR). Combined DCE perfusion parameters (*C*_DCE_) were constructed to predict MVI. The combined model incorporating *C*_DCE_ and CR features was developed and evaluated. Kaplan–Meier method was used to investigate the prognostic significance of the model and the survival benefits of different hepatectomy approaches.

**Results:**

A total of 133 patients were included. Total blood flow in ITR and arterial fraction in PTR exhibited the best predictive performance for MVI with areas under the curve (AUCs) of 0.790 and 0.792, respectively. *C*_DCE_ achieved AUCs of 0.868 (training set) and 0.857 (validation set). A combined model integrated with the α-fetoprotein, corona enhancement, two-trait predictor of venous invasion, and *C*_DCE_ could improve the discrimination ability to AUCs of 0.966 (training set) and 0.937 (validation set). The combined model could stratify the prognosis of HCC patients. Anatomical resection was associated with a better prognosis in the high-risk group (*p* < 0.05).

**Conclusion:**

The combined model integrating DCE perfusion parameters and CR features could be used for MVI prediction in HCC patients and assist clinical decision-making.

**Critical relevance statement:**

The combined model incorporating bi-regional DCE-MRI perfusion parameters and CR features predicted MVI preoperatively, which could stratify the risk of recurrence and aid in optimizing treatment strategies.

**Key Points:**

- Microvascular invasion (MVI) is a significant predictor of prognosis for hepatocellular carcinoma (HCC).
- Quantitative DCE-MRI could predict MVI in solitary BCLC stage A HCC; the combined model improved performance.
- The combined model could help stratify the risk of recurrence and aid treatment planning.

**Graphical Abstract:**

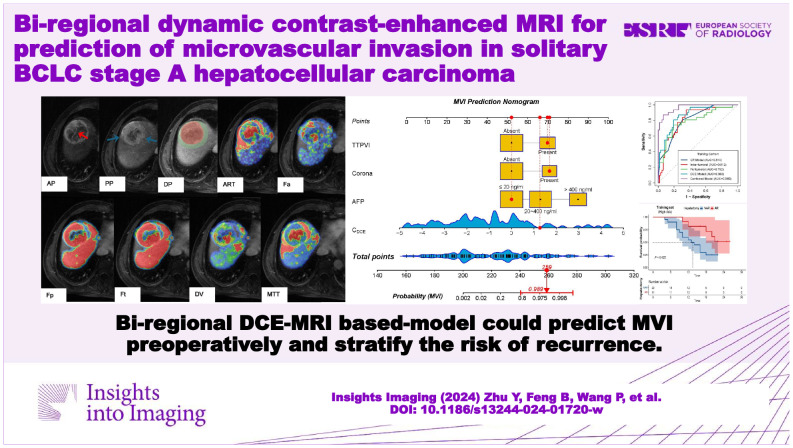

## Introduction

Hepatocellular carcinoma (HCC), the most common type of primary liver malignancy with high aggressiveness, is the sixth most common cancer and the second leading cause of cancer-related deaths globally in 2020 [1]. Radical hepatectomy remains one of the first-line curative therapies in early-stage HCC according to the 2022 Barcelona Clinic Liver Cancer (BCLC) staging classification [2–4]. However, the main drawback of surgical resection is the high recurrence rate of up to 80% [5]. The overall 5-year survival rate of HCC was reported only about 18% [5].

Previous studies have proved that microvascular invasion (MVI) was one of the important risk factors associated with a high recurrence rate and poor prognosis after hepatectomy in HCC [6–8]. The prevalence of MVI was estimated to range from 15 to 57% [6]. MVI was revealed to be an independent predictor of prognosis in BCLC stage A HCC patients but exhibited limited predictive efficiency for individuals with BCLC stages 0 and/or B HCCs [9, 10]. In patients with MVI-positive (MP) HCC, anatomical resection (AR) and wide surgical margins were protective factors for prognosis [11, 12]. In addition, postoperative adjuvant therapy might be potentially beneficial for survival outcomes in patients with concomitant MVI [13]. However, the diagnosis of MVI relies only on the careful examination of postoperative pathological specimens [6–8]. Therefore, efficient identification of MVI status preoperatively is particularly crucial for individualized therapy.

Great efforts have been made to find effective indicators to predict MVI preoperatively based on clinical data and conventional imaging features, such as α-fetoprotein (AFP), tumor size, number, Liver Imaging Reporting and Data System (LI-RADS) version 2018 imaging features, and non-LI-RADS imaging features [14–16]. However, the predictive capacity of a single variable is limited, and morphological evaluation is subjective and experience-dependent. Therefore, it is of great significance to explore a new, combined, accurate, and non-invasive method for predicting MVI.

MR imaging examination is routinely performed for preoperative evaluation of HCC [17]. Recently, quantitative dynamic contrast-enhanced magnetic resonance imaging (DCE-MRI) has emerged as a functional imaging technique through an ultrafast scanning method with high temporal resolution that can capture hemodynamic information of tissue blood flow, microvascular perfusion, and vascular permeability [18, 19]. DCE-MRI could be used for characterizing histopathologic features of various tumors [20–22]. However, DCE-MRI has been applied to HCC in only a few studies due to the nature of the dual blood supply of the liver [22, 23]. Furthermore, during the development of MVI, the peritumoral region (PTR) is the main site of tumor cell invasion. PTR might contain perfusion information different from the intra-tumoral region (ITR), which could reflect the hemodynamic change during the process of MVI. To our knowledge, the perfusion changes of PTR have not been explored where the MVI actually took place. Additionally, whether the prediction model integrating clinical-radiological (CR) features and bi-regional DCE-MRI parameters could stratify the prognosis between HCC patients receiving AR and non-anatomical resection (NAR) has not been explored yet, which might provide evidence for the selection of optimal surgical approaches preoperatively.

In this study, we aimed to develop and validate prediction models for MVI in solitary BCLC stage A HCC based on CR features and DCE-MRI parameters derived from ITR and PTR. Moreover, we compared the therapeutic outcomes of the patients receiving AR or NAR according to the risk stratification for MVI based on the prediction model to identify patients who might benefit from AR.

## Materials and methods

### Study population

**Fig. 1:**
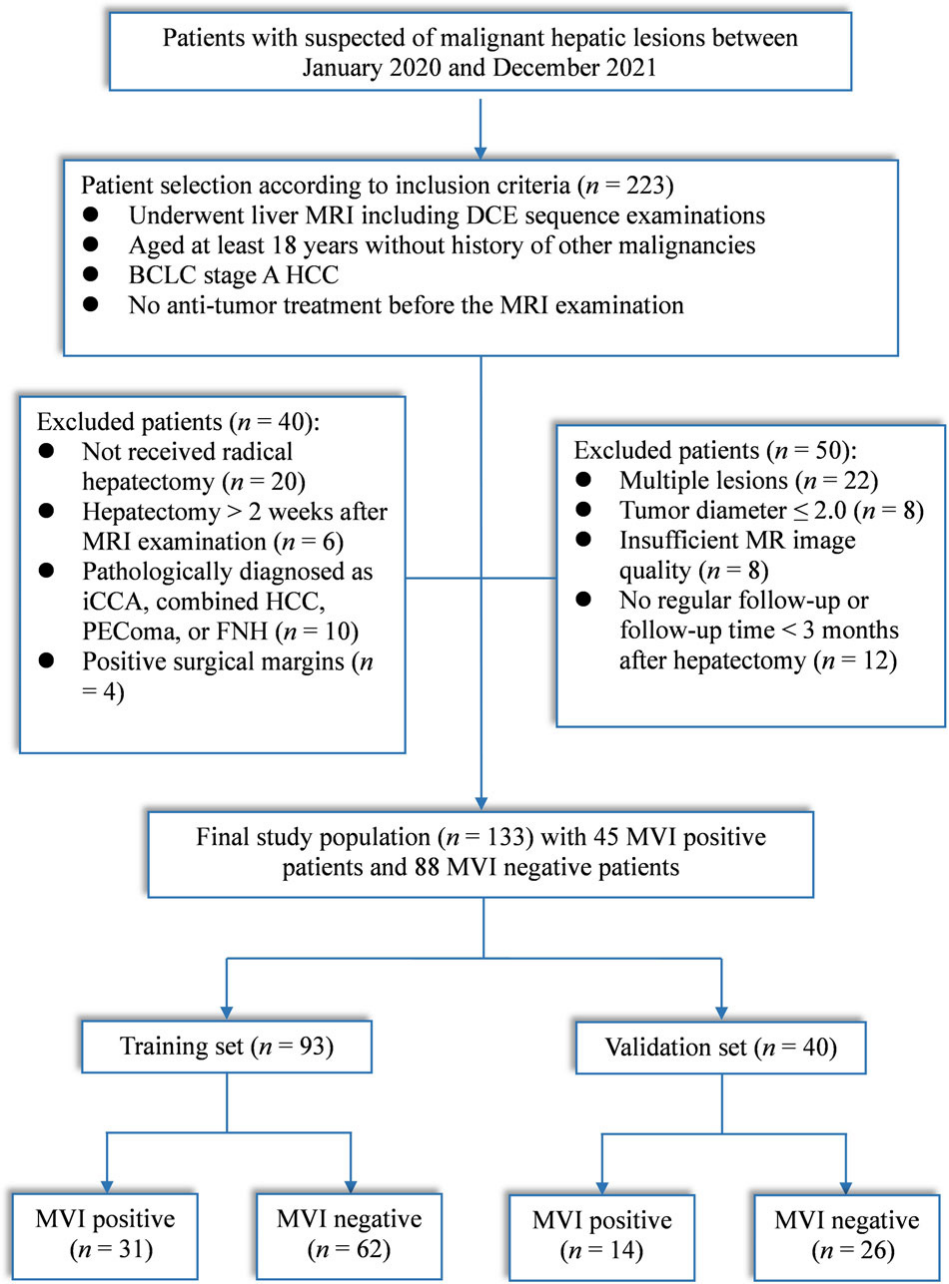
Flowchart of the recruitment of the study population. DCE, dynamic contrast enhanced; iCCA, intrahepatic cholangiocarcinoma; HCC, hepatocellular carcinoma; PEComa, perivascular epithelioid cells tumor; FNH, focal nodular hyperplasia; MVI, microvascular invasion

This single-center prospective study was approved by the Institutional Review Board of the National Cancer Center/Cancer Hospital, Beijing, China (No. 20/412-2608). The written informed consent was obtained from all participants. Between January 2020 and December 2021, consecutive patients underwent MR imaging examinations with quantitative DCE sequence due to suspected malignant hepatic lesions. The inclusion criteria were as follows: (1) over 18 years without other malignancies; (2) BCLC stage A: solitary HCC > 2.0 cm, no obvious macrovascular invasion or extrahepatic spread; (3) no anti-tumor therapeutic intervention. A total of 223 patients met the above criteria initially. 90 patients were excluded due to various reasons (Fig. 1). Finally, 133 patients including 112 men and 21 women with a median age of 57 years (range, 30–73) were included in this study. In order to build the prediction nomogram for MVI, the patients were randomly divided into a training set (*n* = 93) and a validation set (*n* = 40) at a ratio of 7:3 using a random seed method. The flowchart of the study population selection is displayed in Fig. 1.

### MR imaging acquisition

All MR imaging studies were performed with a 3.0-T scanner (SIGNA^TM^ Architect, GE Healthcare, USA) equipped with a 46-channel adaptive image receive body coil. The magnetic resonance imaging (MRI) scan protocols and detailed acquisition parameters of the MRI sequences used are presented in Appendix E1 and Supplementary Table S1. Quantitative DCE-MRI was performed by using liver acquisition with volume acceleration-extended volume sequence according to our previous study [24]. In short, a multiple flip angle method ranging from 3° to 12° was adopted for T1 mapping which was used for the determination of tissue baseline T1 values, and a dynamic enhanced scan with 42 consecutive phases was performed for perfusion quantification.

### DCE MR image processing and analysis

The DCE-MRI was processed using an in-house program written on MATLAB R2018a (MathWorks, Natick, MA, USA). The detailed image post-processing method is described in Appendix E2. The following six pseudo-color maps of the hemodynamic parameters were generated by a voxel-based curve fitting method using all DCE images as input: arterial fraction (ART, %), arterial flow (*F*_a_, mL/min/100 g), portal-venous flow (*F*_p_, mL/min/100 g), total blood flow (*F*_t_, mL/min/100 g), distribution volume (DV, %), and mean transit time (MTT, s).

DCE MR images were analyzed by two independent radiologists (Y.J.Z. with 12 and W.C. with 6 years of experience in abdominal imaging) who were blinded to clinical, pathological, and laboratory information. Freehand-based region of interest (ROI) was manually segmented on the portal-venous phase DCE images using dedicated software (3D Slicer version 5.2.2; https://www.slicer.org/). The intra-tumoral ROI was carefully delineated along the contour of the tumor by referring to T2WI and DWI. Based on a topological algorithm from the tumor margin, the peritumoral ROI was automatically dilated at a radius of 10 mm, and manual correction was performed when the dilated ROI extended beyond the liver boundary. The ROIs were simultaneously copied to DCE perfusion maps and the mean values of DCE parameters in ITR (*F*_a_-T, *F*_p_-T, *F*_t_-T, ART-T, DV-T, and MTT-T) and PTR (*F*_a_-P, *F*_p_-P, *F*_t_-P, ART-P, DV-P, and MTT-P) were automatically extracted. The average measurements from two radiologists were used for the final analysis.

### Conventional MR imaging features

All MR images were reviewed by two board-certified radiologists (S.W. and B.F., with 20 and 10 years of abdominal radiology experience, respectively), who were blinded to the patients’ clinical history and pathological information. Fourteen LI-RADS v2018 imaging features [25] and four non-LI-RADS imaging features (cirrhosis, tumor margin, tumor capsule, and two-trait predictor of venous invasion (TTPVI)) were analyzed. Any discrepancies were settled by consulting a third senior professor (X.H.M., with over 25 years of experience in abdominal imaging), and consensus was reached after discussion. TTPVI was defined as the presence of “internal arteries” in the arterial phase and the absence of continuous “hypodense halos” in the portal-venous or delayed phase [26, 27]. The definitions of each MR imaging feature are described in Supplementary Table S2.

### Clinical characteristics, surgery, and pathological examination

The baseline clinical characteristics were collected from the electronic medical records. All patients underwent AR or NAR radical hepatectomy as appropriate, which was determined by a multidisciplinary team discussion. The MVI status, degree of differentiation, histologic pattern, and fibrosis stage were evaluated and recorded on postoperative specimens.

### Follow-up and endpoints

Patients were followed up regularly at the outpatient clinic every 3 months within 2 years after hepatectomy and every 6 months thereafter, based on AFP and imaging examinations. The study endpoint was recurrence-free survival (RFS). Tumor recurrence was diagnosed according to the criteria of the EASL Clinical Practice Guidelines [17]. The RFS time was defined as the time interval from surgery to the first date of tumor recurrence or the last follow-up before December 31, 2023.

### Statistical analysis and model construction

Variables were compared using independent sample *t*-test, Mann–Whitney *U*-test, *χ*^2^, or Fisher exact test as appropriate. Interobserver variability was assessed by the intraclass correlation coefficient (ICC) or Cohen’s kappa coefficients (*κ*-values) [28]. Multivariate logistic regression was used to identify independent predictors and construct the prediction models. A combined DCE parameter (*C*_DCE_) was generated based on the linear predictors of the regression equation (the sum of the intercept and independent DCE parameters multiplies the regression coefficients). A visualized combined nomogram was established using independent CR features and *C*_DCE_. Finally, the validation set was used to assess the model generalization. The receiving operating curve (ROC) was used to evaluate the predictive performance. Delong test was adopted to compare the predictive performance of ROCs among the models. The Hosmer-Lemeshow goodness-of-fit test and calibration curve were used to evaluate the model’s calibration. Decision curve analysis (DCA) was used to evaluate the clinical value of the combined model. Survival curves of different risk groups and surgical approaches were calculated by the Kaplan–Meier method and compared by log-rank test. A two-tailed *p* < 0.05 was considered statistically significant. All statistical analysis was performed using R software (version 4.2.2; R Foundation, Vienna, Austria).

## Results

### Baseline demographic and clinicopathological characteristics

The clinicopathological characteristics of the patients are described in Table 1. Of the 133 HCC patients enrolled in this study, 33.33% (31/93) in the training set and 35.00% (14/40) in the validation set were categorized into the MP group. The clinicopathological characteristics between the training set and validation set showed no significant difference (all *p* > 0.05). Serum AFP level, HBV DNA load, and histological grade exhibited significant differences between the MP group and the MVI-negative (MN) group (all *p* < 0.05) (Table 1).

**Table 1:** Clinical and pathological characteristics of the study population according to pathological microvascular invasion in the training set and validation set

Variables	Training set (*n* = 93)			Validation set (*n* = 40)			*p-*value^b^
	MP (*n* = 31)	MN (*n* = 62)	*p-*value^a^	MP (*n* = 14)	MN (*n* = 26)	*p*-value^a^	
Clinical characteristics							
Age (years)	56.45 ± 10.05	55.02 ± 10.56	0.525	56.71 ± 9.97	57.89 ± 7.94	0.586	0.564
Gender			1.000			0.399	0.870
Male	26 (83.9)	52 (83.9)		13 (92.9)	21 (80.8)		
Female	5 (16.1)	10 (16.1)		1 (7.1)	5 (19.2)		
AFP (ng/mL)			**0.003**			**0.004**	0.239
≤ 20	5 (16.1)	32 (51.6)		3 (21.4)	19 (73.1)		
20–400	15 (48.4)	20 (32.3)		5 (35.7)	5 (19.2)		
> 400	11 (35.5)	10 (16.1)		6 (42.9)	2 (7.7)		
Location			0.852			1.000	0.690
Right	22 (71.0)	39 (62.9)		9 (64.3)	15 (57.7)		
Left	8 (25.8)	20 (32.3)		5 (35.7)	10 (38.5)		
Caudal	1 (3.2)	3 (4.8)		0 (0)	1 (3.8)		
HBsAg/HCV-Ab			0.918			0.804	0.481
Negative	4 (12.9)	10 (16.1)		2 (14.3)	6 (23.1)		
Positive	27 (87.1)	52 (83.9)		12 (85.7)	20 (76.9)		
HBV DNA load (IU/mL)			**0.034**			**0.037**	0.598
≤ 10^4^	18 (58.1)	49 (79.0)		6 (42.9)	21 (80.8)		
> 10^4^	13 (41.9)	13 (21.0)		8 (57.1)	5 (19.2)		
Antiviral therapy			0.467			0.804	0.955
Yes	5 (16.1)	14 (22.6)		2 (14.3)	6 (23.1)		
No	26 (83.9)	48 (77.4)		12 (85.7)	20 (76.9)		
Hepatectomy			0.877			0.608	0.732
Anatomical	11 (35.5)	21 (33.9)		6 (42.9)	9 (34.6)		
Non-anatomical	20 (64.5)	41 (66.1)		8 (57.1)	17 (65.4)		
Child-Pugh classification			1.000			0.602	1.000
A	28 (90.3)	57 (91.9)		12 (85.7)	24 (92.3)		
B	3 (9.7)	5 (8.1)		2 (14.3)	2 (7.7)		
Platelets ( × 10^9^/L)			0.570			0.646	0.615
≥ 100	26 (83.9)	56 (90.3)		11 (78.6)	23 (88.5)		
< 100	5 (16.1)	6 (9.7)		3 (21.4)	3 (11.5)		
Hemoglobin (g/L)			0.910			0.602	0.995
≥ 120	28 (90.3)	54 (87.1)		12 (85.7)	24 (92.3)		
< 120	3 (9.7)	8 (12.9)		2 (14.3)	2 (7.7)		
Albumin (g/L)			0.598			1.000	0.429
≥ 35	29 (93.5)	60 (96.8)		13 (92.9)	24 (92.3)		
< 35	2 (6.5)	2 (3.2)		1 (7.1)	2 (7.7)		
Glucose (mmol/L)			0.896			0.646	0.427
≤ 7.0	29 (93.5)	56 (90.3)		11 (78.6)	23 (88.5)		
> 7.0	2 (6.5)	6 (9.7)		3 (21.4)	3 (11.5)		
ALT (U/L)			0.652			0.343	0.889
≤ 40	18 (58.1)	39 (62.9)		7 (50.0)	17 (65.4)		
> 40	13 (41.9)	23 (37.1)		7 (50.0)	9 (34.6)		
AST (U/L)			0.881			0.972	0.426
≤ 35	19 (61.3)	37 (59.7)		10 (71.4)	17 (65.4)		
> 35	12 (38.7)	25 (40.3)		4 (28.6)	9 (34.6)		
ALP (U/L)			0.710			0.602	1.000
≤ 100	29 (93.5)	55 (88.7)		12 (85.7)	24 (92.3)		
> 100	2 (6.5)	7 (11.3)		2 (14.3)	2 (7.7)		
GGT (U/L)			0.213			0.677	0.852
≤ 50	18 (58.1)	44 (71.0)		8 (57.1)	18 (69.2)		
> 50	13 (41.9)	18 (29.0)		6 (42.9)	8 (30.8)		
INR			0.660			0.666	0.664
≤ 1.0	14 (45.2)	31 (50.0)		8 (57.1)	13 (50.0)		
> 1.0	17 (54.8)	31 (50.0)		6 (42.9)	13 (50.0)		
PT (s)			0.682			0.640	0.557
≤ 13	28 (90.3)	58 (93.5)		13 (92.9)	22 (84.6)		
> 13	3 (9.7)	4 (6.5)		1 (7.1)	4 (15.4)		
TBil (μmol/L)			0.916			1.000	0.431
≤ 21	26 (83.9)	54 (87.1)		12 (85.7)	22 (84.6)		
> 21	13 (16.1)	8 (12.9)		2 (14.3)	4 (15.4)		
Pathological characteristics							
Edmondson–Steiner grade			**0.004**			**0.037**	0.689
I/II	16 (51.6)	50 (80.6)		6 (42.9)	21 (80.8)		
III/IV	15 (48.4)	12 (19.4)		8 (57.1)	5 (19.2)		
Histologic pattern			0.771			0.907	0.930
Trabecular	7 (22.6)	23 (37.1)		3 (21.4)	8 (30.8)		
Solid	7 (22.6)	13 (21.0)		3 (21.4)	4 (15.4)		
Pseudoglandular	3 (9.7)	4 (6.5)		1 (7.1)	3 (11.5)		
Macrotrabecular	5 (16.1)	8 (12.9)		3 (21.4)	3 (11.5)		
Others	2 (6.5)	4 (6.5)		1 (7.1)	1 (3.8)		
Mixed	7 (22.6)	10 (16.1)		3 (21.4)	7 (26.9)		
Fibrosis stage			0.658			0.842	0.986
S1-S2	13 (41.9)	29 (46.8)		6 (42.9)	12 (46.2)		
S3-S4	18 (58.1)	33 (53.2)		8 (57.1)	14 (53.8)		

Data are expressed as mean ± SD or number with percentage in parentheses. Statistically significant results (*p* < 0.05) are marked in bold

*AFP* alpha-fetoprotein, *ALP* alkaline phosphatase, *ALT* alanine aminotransferase, *AST* aspartate aminotransaminase, *BCLC* Barcelona Clinic Liver Cancer, *GGT* gamma-glutamyl transpeptidase, *HBsAg/HCV-Ab* hepatitis B virus surface antigen/hepatitis C virus antibodies, *HBV* hepatitis B virus, *INR* international normalized ratio, *MN* microvascular invasion negative, *MP* microvascular invasion positive, *TBil* total bilirubin

^a^ Comparison between the MP group and the MN group

^b^ Comparison between the training set and validation set

### Interobserver agreement

The interobserver agreement analysis of radiological features and quantitative DCE parameters are shown in Supplementary Tables S3 and S4. Agreement between the two observers was good to excellent, with kappa values of 0.674–1.000 for radiological features and ICC values of 0.804–0.953 for quantitative DCE parameters.

### Radiological characteristics

The radiological features of the patients are shown in Table 2. In the MP group, the following five MR imaging characteristics were more frequently observed compared to the MN group: mosaic architecture, corona enhancement, non-smooth tumor margin, incomplete tumor capsule, and TTPVI, both in the training and validation sets (all *p* < 0.05).

**Table 2:** Radiological characteristics of the study population according to pathological microvascular invasion in the training set and validation set

Variables	Training set (*n* = 93)			Validation set (*n* = 40)			*p-*value^b^
	MP (*n* = 31)	MN (*n* = 62)	*p-*value^a^	MP (*n* = 14)	MN (*n* = 26)	*p-*value^a^	
LI-RADS major features							
Tumor size (cm)			…			…	…
< 2.0	0 (0)	0 (0)		0 (0)	0 (0)		
≥ 2.0	31 (100)	62 (100)		14 (100)	26 (100)		
Non-rim arterial phase hyperenhancement			0.598			0.533	1.000
Absent	2 (6.5)	2 (3.2)		0 (0)	2 (7.7)		
Present	29 (93.5)	60 (96.8)		14 (100)	24 (92.3)		
Non-peripheral washout			0.716			0.694	0.788
Absent	7 (22.6)	12 (19.4)		4 (28.6)	5 (19.2)		
Present	24 (77.4)	50 (80.6)		10 (71.4)	21 (80.8)		
Enhancing capsule			0.537			0.864	0.732
Absent	12 (38.7)	20 (32.3)		5 (35.7)	10 (38.5)		
Present	19 (61.3)	42 (67.7)		9 (64.3)	16 (61.5)		
LI-RADS ancillary features (favoring HCC in particular)							
Non-enhancing capsule			0.906			0.646	0.850
Absent	27 (87.1)	56 (90.3)		11 (78.6)	23 (88.5)		
Present	4 (12.9)	6 (9.7)		3 (21.4)	3 (11.5)		
Nodule-in-nodule architecture			0.397			0.602	0.750
Absent	28 (90.3)	59 (95.2)		12 (85.7)	24 (92.3)		
Present	3 (9.7)	3 (4.8)		2 (14.3)	2 (7.7)		
Mosaic architecture			**0.007**			**0.021**	0.889
Absent	13 (41.9)	44 (71.0)		5 (35.7)	19 (73.1)		
Present	18 (58.1)	18 (29.0)		9 (64.3)	7 (26.9)		
Fat in mass, more than adjacent liver			0.734			0.795	0.737
Absent	24 (77.4)	46 (74.2)		11 (78.6)	18 (69.2)		
Present	7 (22.6)	16 (25.8)		3 (21.4)	8 (30.8)		
Blood products in mass			0.730			0.828	0.442
Absent	23 (74.2)	48 (77.4)		9 (64.3)	19 (73.1)		
Present	8 (25.8)	14 (22.6)		5 (35.7)	7 (26.9)		
LI-RADS ancillary features (favoring malignancy, not HCC in particular)							
Restricted diffusion			…			…	…
Absent	0 (0)	0 (0)		0 (0)	0 (0)		
Present	31 (100)	62 (100)		14 (100)	26 (100)		
Mild-moderate T2 hyperintensity			…			…	…
Absent	0 (0)	0 (0)		0 (0)	0 (0)		
Present	31 (100)	62 (100)		14 (100)	26 (100)		
Corona enhancement			**0.001**			**0.012**	0.578
Absent	15 (48.4)	50 (80.6)		5 (35.7)	21 (80.8)		
Present	16 (51.6)	12 (19.4)		9 (64.3)	5 (19.2)		
Fat sparing in the solid mass			1.000			1.000	0.895
Absent	29 (93.5)	57 (91.9)		13 (92.9)	23 (88.5)		
Present	2 (6.5)	5 (8.1)		1 (7.1)	3 (11.5)		
Iron sparing in solid mass			0.598			0.602	0.384
Absent	29 (93.5)	60 (96.8)		12 (85.7)	24 (92.3)		
Present	2 (6.5)	2 (3.2)		2 (14.3)	2 (7.7)		
Non-LI-RADS imaging features							
Cirrhosis			0.660			0.641	0.720
Absent	15 (48.4)	33 (53.2)		7 (50.0)	15 (57.7)		
Present	16 (51.6)	29 (46.8)		7 (50.0)	11 (42.3)		
Tumor margin			**0.015**			**0.037**	0.572
Smooth	14 (45.2)	44 (71.0)		6 (42.9)	21 (80.8)		
Non-smooth	17 (54.8)	18 (29.0)		8 (57.1)	5 (19.2)		
Tumor capsule			**0.024**			**0.026**	0.895
Complete	14 (45.2)	43 (69.4)		5 (35.7)	20 (76.9)		
Incomplete/absent	17 (54.8)	19 (30.6)		9 (64.3)	6 (23.1)		
TTPVI			**0.019**			**0.049**	0.586
Absent	16 (51.6)	47 (75.8)		7 (50.0)	22 (84.6)		
Present	15 (48.4)	15 (24.2)		7 (50.0)	4 (15.4)		

Data are expressed as numbers with percentages in parentheses. Statistically significant results (*p* < 0.05) are marked in bold. Ellipses indicated all observations (133/133) showed the feature of greater than 2.0 cm, restricted diffusion, and mild-moderate T2 hyperintensity

*HCC* hepatocellular carcinoma, *LI-RADS* Liver Imaging Reporting, and Data System, *MN* microvascular invasion negative, *MP* microvascular invasion positive, *TTPVI* a two-trait predictor of venous invasion

^a^ Comparison between the MP group and the MN group

^b^ Comparison between the training set and validation set

### Quantitative DCE-MRI parameters and the predictive performance of MVI

**Fig. 2:**
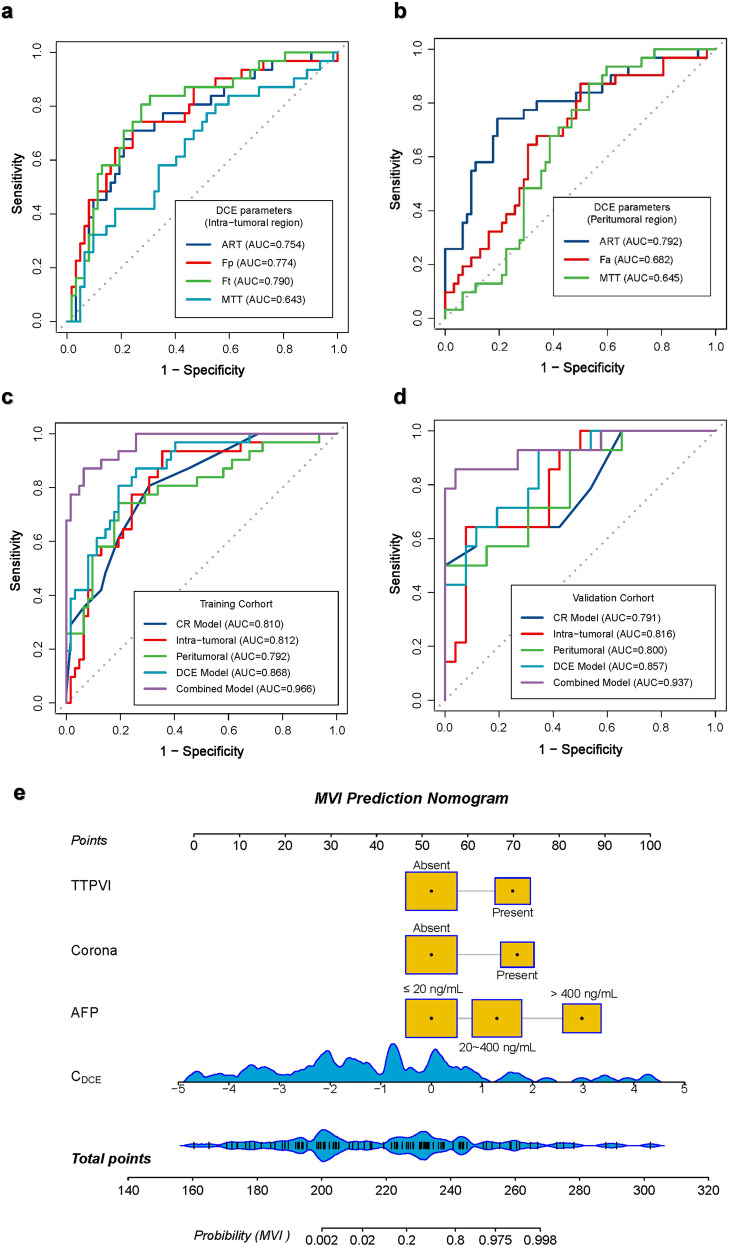
ROC curves of the DCE parameters and prediction models, and the combined prediction nomogram in the prediction of MVI status of HCC. **a** Predictive performance of intra-tumoral DCE parameters in the training set. **b** Predictive performance of peritumoral DCE parameters in the training set. **c** ROC curves of the CR, intra-tumoral, peritumoral, DCE, and combined models in the training set. **d** ROC curves of the clinical, intra-tumoral, peritumoral, DCE, and combined models in the validation set. Both in the training set and the validation set, the combined model showed the best prediction performance. **e** A nomogram combines three independent CR predictors (corona enhancement, TTPVI, and serum AFP) and a combined DCE parameter (*C*_DCE_). The cutoff point of our nomogram in terms of total points is 232.3 points. AFP, alpha-fetoprotein; ART, arterial fraction; CR, clinic-radiological; DCE, dynamic contrast enhanced; *F*_a_, arterial blood flow; *F*_p_, portal-venous blood flow; *F*_t_, total blood flow; MTT, mean transit time; MVI, microvascular invasion; TTPVI, two-trait predictor of venous invasion

The results of the comparison of quantitative DCE-MRI parameters between MP and MN groups are summarized in Table 3 and Supplementary Figs. 1S–4S. In the ITR, *F*_p_-T and *F*_t_-T were significantly higher, while ART-T and MTT-T were significantly lower in the MP group than in the MN group (all *p* < 0.05). In the PTR, ART-P and *F*_a_-P were significantly higher, while MTT-P were significantly lower in the MP group than the MN group (all *p* < 0.05). ROC analyses of significant parameters in the training set were described in Table 4 and Fig. 2a, b. Among these single parameters, *F*_t_-T and ART-P exhibited the best predictive performance for discriminating MVI status, with AUCs of 0.790 and 0.792, respectively.

**Table 3:** Comparison of DCE quantitative parameters between different microvascular invasion statuses in the training set and validation set

Parameters	Training set (*n* = 93)			Validation set (*n* = 40)		
	MP (*n* = 31)	MN (*n* = 62)	*p-*value^a^	MP (*n* = 14)	MN (*n* = 26)	*p-*value^a^
Intra-tumoral region						
ART (%)	53.85 (47.81, 62.34)	66.97 (59.81, 74.58)	**<** **0.001**	53.64 (48.43, 59.99)	66.21 (55.86, 69.41)	**0.003**
* F*_a_ (mL/min/100 g)	104.24 (92.31, 123.74)	113.44 (92.18, 130.02)	0.444	107.78 (91.50, 115.06)	109.37 (100.46, 120.42)	0.440
* F*_p_ (mL/min/100 g)	83.68 (62.95, 105.33)	57.35 (45.64, 71.40)	**<** **0.001**	87.57 (73.45, 104.89)	59.99 (51.69, 77.59)	**<** **0.001**
* F*_t_ (mL/min/100 g)	197.30 (178.05, 215.47)	169.94 (155.11, 186.15)	**<** **0.001**	205.45 (181.50, 227.82)	169.08 (153.49, 196.78)	**0.031**
DV (%)	23.17 (20.00, 27.76)	22.62 (20.65, 25.12)	0.234	24.20 (23.09, 28.52)	23.09 (19.84, 30.68)	0.279
MTT (s)	12.12 (8.84, 13.21)	13.09 (11.30, 15.10)	**0.025**	12.39 (9.00, 13.88)	14.31 (12.43, 16.65)	**0.029**
Peritumoral region						
ART (%)	30.41 (26.01, 37.80)	24.45 (20.96, 27.94)	**<** **0.001**	30.40 (25.48, 33.85)	23.74 (20.84, 27.27)	**0.001**
* F*_a_ (mL/min/100 g)	50.69 (39.52, 62.36)	37.38 (27.59, 54.96)	**0.004**	50.71 (39.14, 79.25)	35.26 (25.81, 45.95)	**0.006**
* F*_p_ (mL/min/100 g)	106.20 (83.77, 149.83)	114.98 (92.45, 157.45)	0.340	105.25 (78.58, 140.31)	109.33 (85.64, 141.75)	0.630
* F*_t_ (mL/min/100 g)	156.04 (120.80, 212.65)	158.56 (121.92, 214.90)	0.871	152.43 (109.27, 201.31)	143.51 (118.43, 187.52)	0.755
DV (%)	20.89 (16.02, 24.03)	21.54 (16.10, 24.21)	0.237	20.85 (19.73, 31.72)	23.34 (21.33, 26.11)	0.799
MTT (s)	19.62 (16.76, 20.79)	20.89 (17.04, 23.58)	**0.023**	18.33 (14.82, 21.43)	21.26 (18.29, 25.73)	**0.036**

Data are given as median (inter-quartile ranges). Statistically significant results (*p* < 0.05) are marked in bold

*ART* arterial fraction, *DCE* dynamic contrast enhanced, *DV* distribution volume, *F*_*a*_ arterial blood flow, *F*_*p*_ portal-venous blood flow, *F*_*t*_ total blood flow, *MN* MVI negative, *MP* MVI positive, *MTT* mean transit time, *MVI* microvascular invasion

^a^ *p*-value represents a comparison between the MP group and the MN group and was calculated with the Mann–Whitney *U*-test

**Table 4:** Predictive efficacy of DCE quantitative parameters for MVI status of HCC in the training set

Parameters	Threshold	AUC (95% CI)	Accuracy (%)	Sensitivity (%)	Specificity (%)	PPV (%)	NPV (%)
Intra-tumoral region							
ART-T (%)	58.35	0.754 (0.648, 0.860)	75.27 (70/93)	67.74 (21/31)	79.03 (49/62)	61.76 (21/34)	83.05 (49/59)
* F*_p_-T (mL/min/100 g)	70.85	0.774 (0.670, 0.878)	75.27 (70/93)	74.19 (23/31)	75.81 (47/62)	60.53 (23/38)	85.45 (47/55)
* F*_t_-T (mL/min/100 g)	180.71	0.790 (0.692, 0.888)	75.27 (70/93)	80.65 (25/31)	72.58 (45/62)	59.52 (25/42)	88.24 (45/51)
MTT-T (s)	13.28	0.643 (0.521, 0.765)	58.06 (54/93)	77.42 (24/31)	48.39 (30/62)	42.86 (24/56)	81.08 (30/37)
Peritumoral region							
ART-P (%)	28.19	0.792 (0.690, 0.895)	78.49 (73/93)	74.19 (23/31)	80.65 (50/62)	65.71 (23/35)	86.21 (50/58)
* F*_a_-P (mL/min/100 g)	36.36	0.682 (0.569, 0.795)	62.37 (58/93)	87.10 (27/31)	50.00 (31/62)	46.55 (27/58)	88.57 (31/35)
MTT-P (s)	21.10	0.645 (0.534, 0.755)	60.22 (66/93)	87.10 (27/31)	46.77 (29/62)	45.00 (27/60)	87.88 (29/33)

Except for AUC, values are percentages with the number of examinations in parentheses

*ART* arterial fraction, *AUC* area under the curve, *CI* confidence interval, *DCE* dynamic contrast enhanced, *F*_*a*_ arterial blood flow, *F*_*p*_ portal-venous blood flow, *F*_*t*_ total blood flow, *HCC* hepatocellular carcinoma, *MTT* mean transit time, *MVI* microvascular invasion, *NPV* negative predictive value, *P* peritumoral region, *PPV* positive predictive value, *T* intra-tumoral region

### Construction and validation of the MVI prediction models

The significant features were used as covariates to construct CR, intra-tumoral, peritumoral, DCE, and combined prediction models through multivariate logistic analysis (Supplementary Table S5). The results showed that AFP, corona enhancement, and TTPVI were independent risk factors for MVI. The combined DCE-MRI parameters (*C*_DCE_) could be calculated as follows: *C*_DCE_ = −10.052 + 0.026 × *F*_t_ − *T* + 0.170 × ART-P.

A combined prediction model was built using AFP, corona enhancement, TTPVI, and *C*_DCE_ through multivariate logistic regression (Supplementary Table S5). The predictive performance of models is described in Table 5 and Fig. 2c, d. The AUC of the combined model was significantly higher than other models (Delong test, all *p* < 0.05) (Supplementary Fig. S5), which was 0.966 in the training set and 0.937 in the validation set, respectively.

**Table 5:** Predictive efficacy of clinical, DCE, and combined model for MVI status of hepatocellular carcinoma

Models	AUC (95% CI)	Accuracy (%)	Sensitivity (%)	Specificity (%)	PPV (%)	NPV (%)
Training set						
CR model	0.810 (0.722, 0.898)	73.12 (68/93)	80.65 (25/31)	69.35 (43/62)	56.82 (25/44)	87.76 (43/49)
Intra-tumoral model	0.812 (0.719, 0.905)	74.19 (69/93)	93.55 (29/31)	64.52 (40/62)	58.86 (29/51)	95.24 (40/42)
Peritumoral model	0.792 (0.690, 0.895)	78.49 (73/93)	74.19 (23/31)	80.65 (50/62)	65.71 (23/35)	86.21 (50/58)
DCE model	0.868 (0.795, 0.942)	80.65 (75/93)	80.65 (25/31)	80.65 (50/62)	67.57 (25/37)	89.29 (50/56)
Combined model	0.966 (0.935, 0.997)	91.40 (85/93)	87.10 (27/31)	93.55 (58/62)	87.10 (27/31)	93.55 (58/62)
Validation set						
CR model	0.791 (0.773, 0.994)	72.50 (29/40)	78.57 (11/14)	69.23 (18/26)	57.89 (11/19)	85.71 (18/21)
Intra-tumoral model	0.816 (0.681, 0.951)	67.50 (27/40)	78.57 (11/14)	61.54 (16/26)	52.38 (11/21)	84.21 (16/19)
Peritumoral model	0.800 (0.654, 0.945)	75.00 (30/40)	57.14 (8/14)	84.62 (22/26)	66.67 (8/12)	78.57 (22/28)
DCE model	0.857 (0.740, 0.974)	77.50 (31/40)	71.43 (10/14)	80.77 (21/26)	66.67 (10/15)	84.00 (21/25)
Combined model	0.937 (0.847, 1.000)	92.50 (37/40)	85.71 (12/14)	96.15 (25/26)	92.31 (12/13)	92.59 (25/27)

*AUC* area under the curve, *CI* confidence interval, *CR* clinic-radiological, *DCE* dynamic contrast enhanced, *MVI* microvascular invasion, *NPV* negative predictive value, *PPV* positive predictive value

**Fig. 3:**
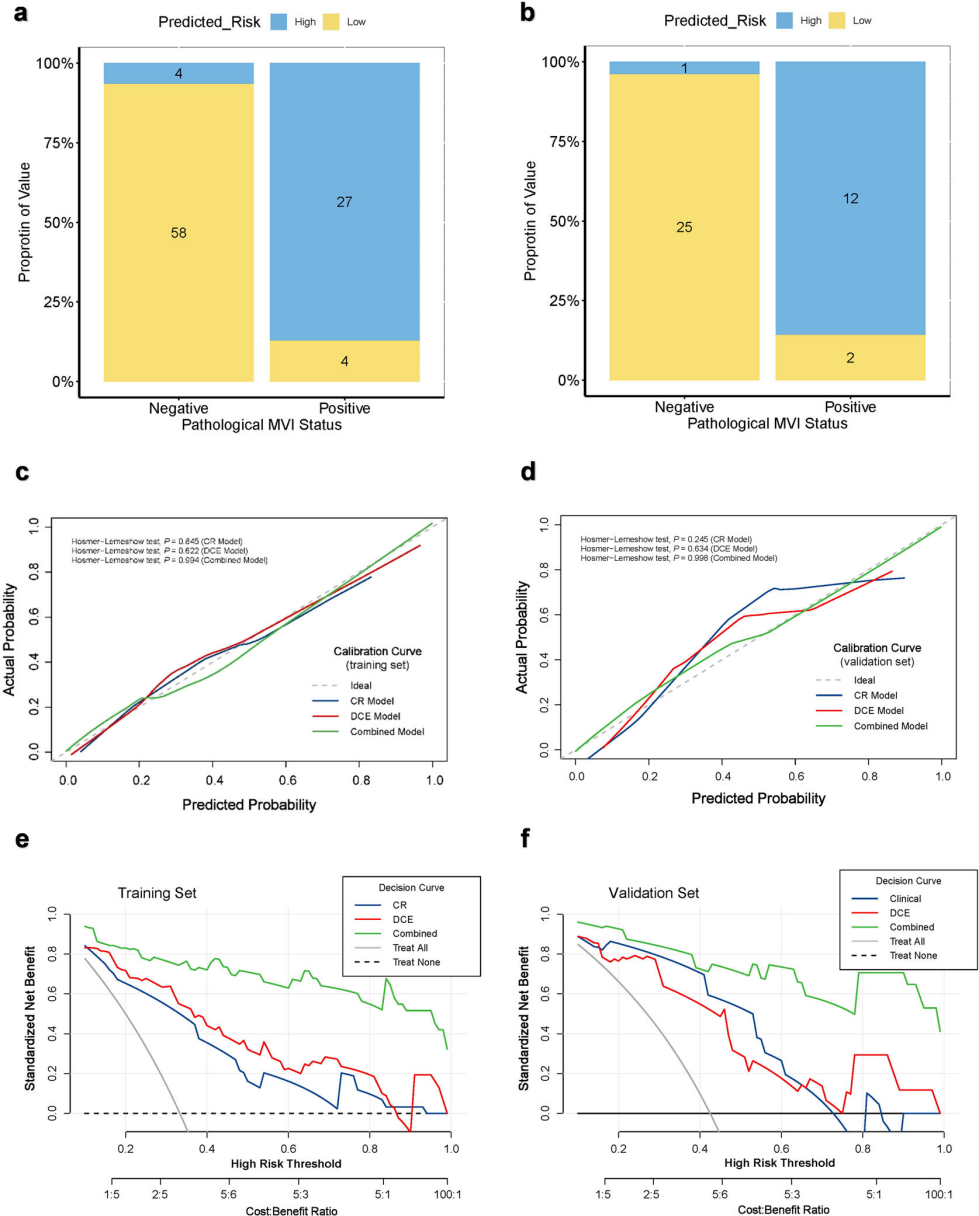
Evaluation and validation of the prediction models. **a**, **b** Bar chart of discrimination performance of combined nomogram for MVI status of HCC in the training set (**a**) and validation set (**b**). The blue box showed the predicted MVI high-risk, and the yellow box showed the predicted MVI low-risk. **c**, **d** Calibration curves of the CR, DCE, and combined models in the training set (**c**) and validation set (**d**). The *x*-axis represents a nomogram-estimate MVI risk, the *y*-axis represents the actual MVI risk, and the diagonal dashed line indicates the ideal prediction by an ideal model. The results of the Hosmer-Lemeshow test were shown in the upper left corner, indicating no significant difference (all *p* > 0.05). **e**, **f** Decision curves of the CR, DCE, and combined models in the training set (**e**) and validation set (**f**). The gray curve represents the assumption that all patients with positive MVI, and the horizontal black dashed line represents the assumption that no patient with positive MVI. CR, clinic-radiological; DCE, dynamic contrast enhanced; MVI, microvascular invasion

A nomogram based on the combined model was constructed to facilitate predicting MVI (Fig. 2e). A risk score for MVI based on the nomogram could be calculated as follows: risk score = 208 + 14 × AFP (20–400 ng/mL) or 33 × AFP (> 400 ng/mL) + 19 × corona enhancement (present) + 18 × TTPVI (present) + 11 × *C*_DCE_. Patients could be dichotomized into high-risk MVI (HMVI, RS > 232.3) or low-risk MVI (LMVI, RS ≤ 232.3) groups based on the RS threshold from ROC analysis (Fig. 3a, b).

**Fig. 4:**
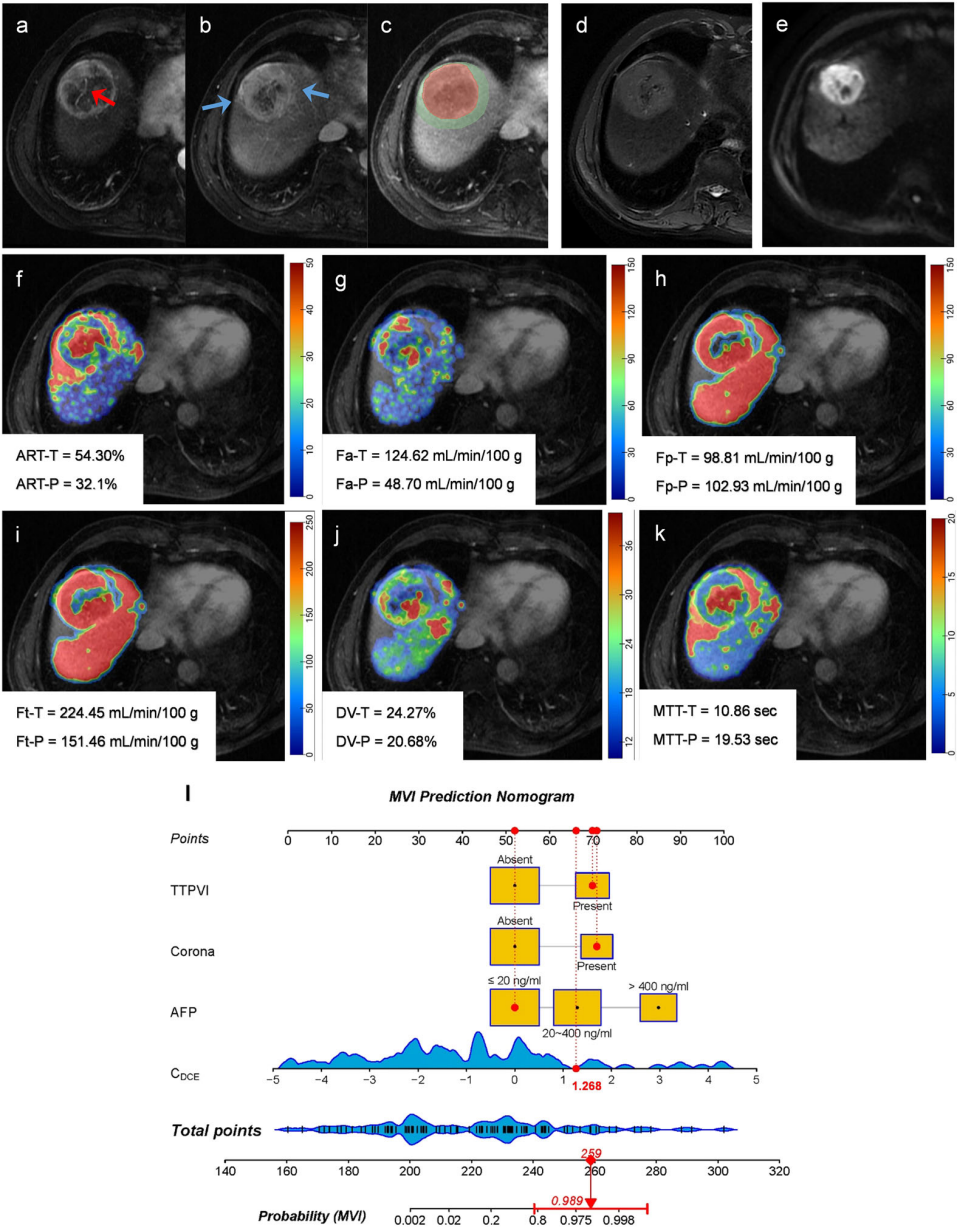
MR images of a 53-year-old male with MVI-positive HCC. Serum AFP was 12.0 ng/mL. **a**–**e** DCE MR images of arterial phase (**a**), portal-venous phase (**b**), delayed phase (**c**), T2-weighted (**d**), and diffusion weighted (**e**), respectively. A 5.0 cm mass with hyperenhancement in the arterial phase, moderate-high signal in T2-weighted images, and diffusion restriction was located on segment VIII of the liver. The red arrow shows the internal arteries inside the tumor in the arterial phase, without a hypointense halo in a post-arterial phase, indicating TTPVI positive. The blue arrow shows the corona enhancement present in the peritumoral region. The segmentation of the tumor and peritumoral region was shown in red and green shadows (**c**). **f**–**k** DCE quantitative parameter pseudo-color maps of ART, *F*_a_, *F*_p_, *F*_t_, DV, and MTT with mean values indicated in the figures, respectively. **l** The utilization of the nomogram to predict the risk of MVI. The corresponding score of each variable is seen on the Points scale. When point scores for all variables were added, total scores and the corresponding probability of MVI were presented on total points and probability scales, respectively. Moreover, observation values are superimposed on the plot and are shown as red dots and solid or dashed droplines. The *C*_DCE_ value of this patient was 1.268. After points for each predictor were added, the total points were 259. The corresponding risk of MVI was 0.989. Histologic examination verified MVI-positive status. AFP, alpha-fetoprotein; ART, arterial fraction; DCE, dynamic contrast enhanced; DV, distribution volume; *F*_a_, arterial blood flow; *F*_p_, portal-venous blood flow; *F*_t_, total blood flow; MTT, mean transit time; MVI, microvascular invasion; TTPVI, two-trait predictor of venous invasion

**Fig. 5:**
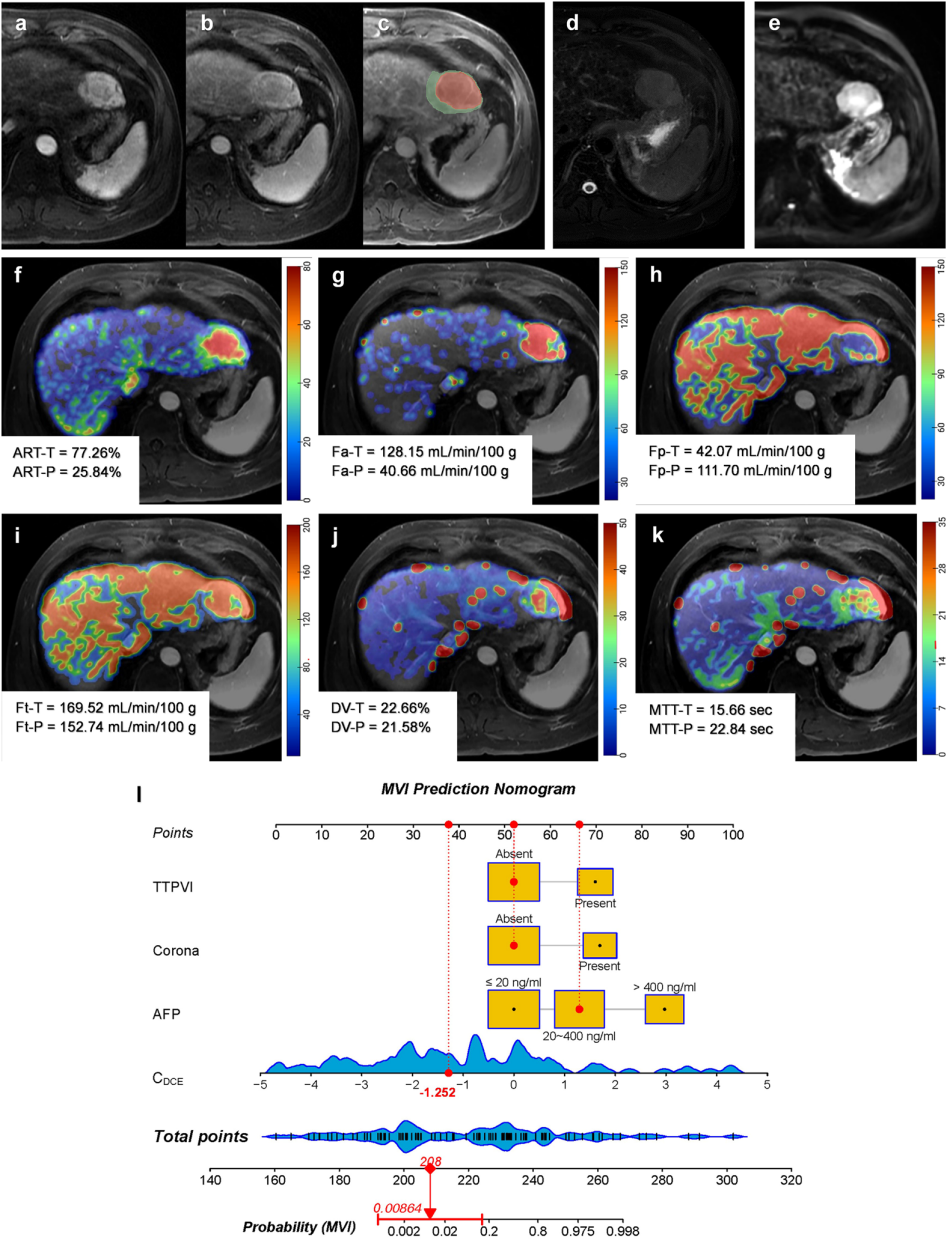
MR images of a 48-year-old male with MVI-negative HCC. Serum AFP was 95.0 ng/mL. **a**–**e** DCE MR images of arterial phase (**a**), portal-venous phase (**b**), delayed phase (**c**), T2-weighted (**d**), and diffusion weighted (**e**), respectively. A 4.6 cm mass with hyperenhancement in the arterial phase, the moderate-high signal in T2-weighted images, and diffusion restriction were located on segment II of the liver. Neither corona enhancement nor TTPVI could be observed. The segmentation of the tumor and peritumoral region was shown in red and green shadows (**c**). **f**–**k** DCE quantitative parameter pseudo-color maps of ART, *F*_a_, *F*_p_, *F*_t_, DV, and MTT with mean values indicated in the figures, respectively. **l** The utilization of the nomogram to predict the risk of MVI. The corresponding score of each variable is seen on the Points scale. When point scores for all variables were added, total scores and the corresponding probability of MVI were presented on total points and probability scales, respectively. Moreover, observation values are superimposed on the plot and are shown as red dots and solid or dashed droplines. The *C*_DCE_ value of this patient was −1.252. After points for each predictor were added, the total points were 208. The corresponding risk of MVI was 0.009. Histologic examination verified MVI-negative status. AFP, alpha-fetoprotein; ART, arterial fraction; DCE, dynamic contrast enhanced; DV, distribution volume; *F*_a_, arterial blood flow; *F*_p_, portal-venous blood flow; *F*_t_, total blood flow; MTT, mean transit time; MVI, microvascular invasion; TTPVI, two-trait predictor of venous invasion

The calibration curve and Hosmer-Lemeshow test (all *p* > 0.05) showed that all prediction models had good agreement with the model fit (Fig. 3c, d). The DCA curve demonstrated that the clinical net benefit of the combined prediction model was almost higher than the CR and DCE models (Fig. 3e, f). The application of the combined model and nomogram is shown in Figs. 4 and  5.

### Recurrence risk stratification according to the combined nomogram and surgical approaches

**Fig. 6:**
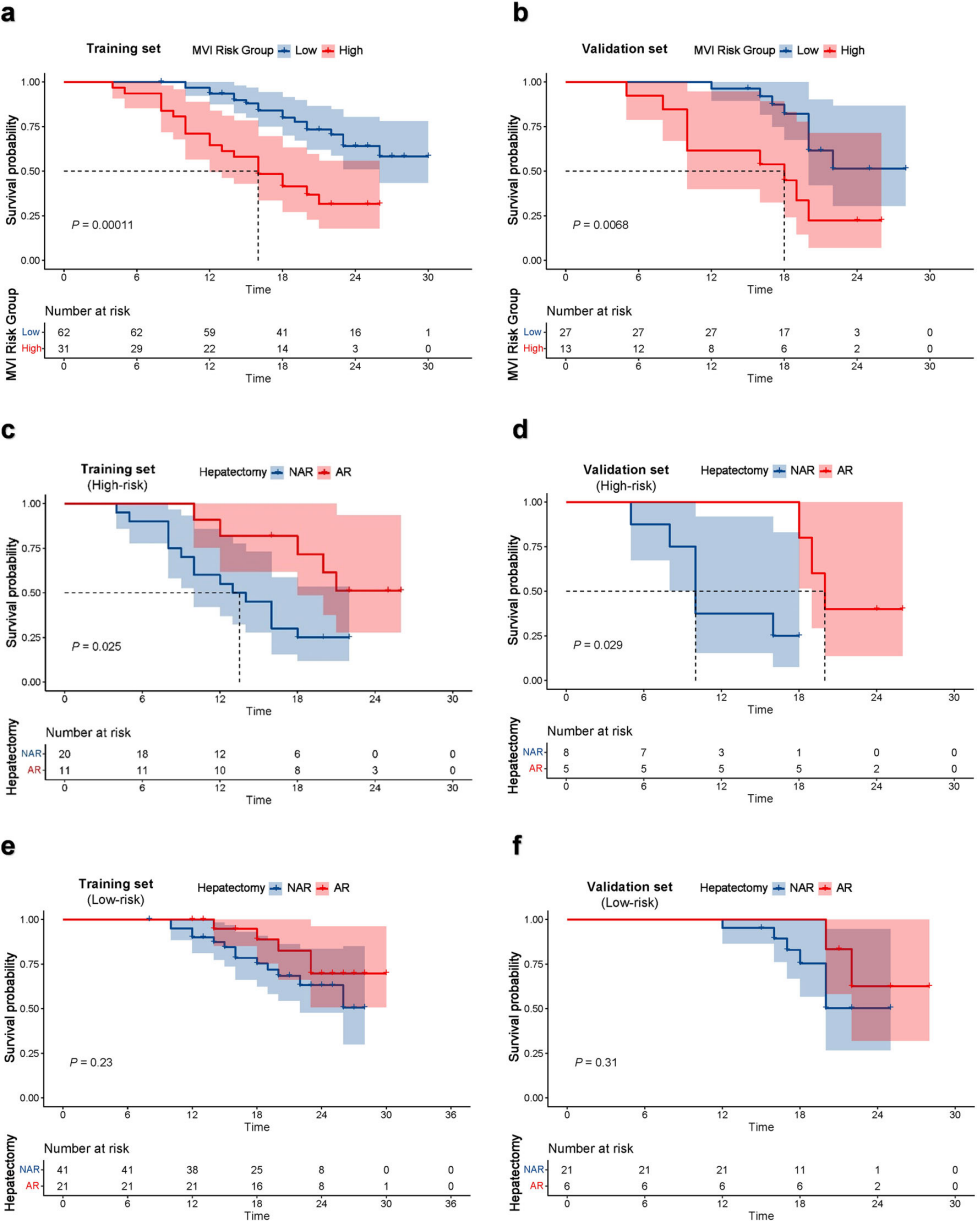
Kaplan–Meier survival curve of recurrence-free survival (RFS) in HCC patients after hepatectomy in the training set (**a**, **c**, **e**) and validation set (**b**, **d**, **f**). **a**, **b** Survival risk stratification was grouped by the combined nomogram. The result showed the nomogram had significant capability for risk stratification (*p* < 0.05), both in the training set (**a**) and the validation set (**b**). **c**, **d** Kaplan–Meier survival curves comparing AR with NAR in patients with MVI high-risk. Patients receiving AR showed a significant higher RFS rate compared with those receiving NAR (*p* < 0.05), both in the training set (**c**) and the validation set (**d**). **e**, **f** Kaplan–Meier survival curves comparing AR with NAR in patients with MVI low-risk. No significant differences were observed regarding RFS between the patients receiving AR and NAR (*p* > 0.05), both in the training set (**e**) and the validation set (**f**). AR, anatomical resection; HCC, hepatocellular carcinoma; MVI, microvascular invasion; NAR, non-anatomical resection

The median follow-up period was 20 (range: 4–35) months. The median RFS of the HMVI group was significantly shorter (training set: 16 months vs. over 30 months; validation set: 18 months vs. over 28 months), and the 2-year RFS rate was significantly lower (training set: 31.6% vs. 64.1%; validation set: 22.4% vs. 51.4%) than that of LMVI group (both *p* < 0.05) (Fig. 6a, b).

In terms of clinical benefit from different surgical approaches, patients with HMVI who received AR exhibited a better prognosis than those who received NAR (training set: *p* = 0.025; validation set: *p* = 0.029) (Fig. 6c, d). However, no significant difference between AR and NAR was observed in patients with LMVI (training set: *p* = 0.230; validation set: *p* = 0.310) (Fig. 6e, f).

## Discussion

In this present study, we successfully developed and validated a combined nomogram based on CR features and quantitative DCE parameters that could predict the MVI status of solitary BCLC stage A preoperatively. The AUC values of the nomogram achieved 0.966 and 0.937 in the training and validation sets, respectively. In addition, our nomogram successfully stratified HCC patients in terms of RFS after hepatectomy based on the dichotomized MVI-risk groups. Moreover, the patients of the HMVI group receiving AR demonstrated a better prognosis than those receiving NAR.

Previous studies indicated that clinical characteristics and conventional imaging features were valuable for MVI prediction [14–16]. Our results revealed that AFP, corona enhancement, and TTPVI were independent predictors of MVI. AFP and corona enhancement could serve as indicators for tumor load and abnormal venous draining, which showed potential capacity in predicting MVI [25, 29]. The tumor might destroy intra-tumoral hepatic veins and later compress peritumoral hepatic veins when MVI occurs, altering the venous drainage and leading to the occurrence of corona enhancement. TTPVI is a diagnostic algorithm showing the ability in the prediction of MVI based on two imaging signs (internal arteries and hypointense halos) [27]. We found the CR model could predict MVI with an AUC of 0.791.

To the best of our knowledge, this is the first study to investigate the hemodynamic perfusion changes in both ITR and PTR for predicting MVI status in HCC. Our results revealed that *F*_p_-T and *F*_t_-T were significantly higher, while ART-T and MTT-T were significantly lower in the MP group than in the MN group. During the progression of HCC, hepatic artery flow and portal-venous flow of the tumor experience a series of complicated changes [30, 31]. The increased *F*_t_-T in HCC might be caused by endothelial cell proliferation and tumor angiogenesis, which could increase total blood perfusion and promote the formation of MVI. We hypothesized the increase of *F*_p_-T might be caused by hepatic artery-portal vein fistula formation and arterialization of the portal vein. Thus, the blood could bypass the capillary network and enter the hepatic vein directly, causing an increase in *F*_p_-T as well as a decrease in ART-T and MTT-T. Tumor cell clusters might be brought into the hepatic venules during this perfusion alteration. There was also evidence that suggested the arterial blood supply of HCC significantly decreased as the histologic grade increased [32]. PTR is the main site where MVI occurs and contains perfusion information that can reflect the hemodynamic change during MVI [6–8], which has drawn increased attention from researchers recently [33, 34]. The increased hepatic arterial perfusion compensatory for reduced portal-venous perfusion, which is brought on by microscopic tumor thrombi in PTR obstructing small portal-venous branches, might account for the elevated ART-P and *F*_a_-P. A *C*_DCE_ (*F*_t_-T and ART-P) was conducted to predict MVI with an AUC of 0.857.

This combined nomogram predicted the MVI status of solitary BCLC stage A HCC with excellent predictive performance. The RFS of HMVI was significantly shorter than that of LMVI. Given the fact that over half of HCC may suffer from tumor recurrence after hepatectomy [5], it is crucial to take measures to prevent recurrence. Emerging evidence has demonstrated that AR was associated with a survival benefit, but AR required a more conservative liver function reserve [35–38]. Therefore, identifying the patients who are suitable for and could benefit from AR is an important issue in the clinic. Our result showed AR significantly reduced the recurrence rate in the HMVI group, but no significance was observed in the LMVI group. For patients at high risk for MVI, implementing AR might yield greater survival benefits if the liver function reserve is adequate. The combined nomogram may be an important indicator for HCC recurrence evaluation and an initial basis for selecting a personalized treatment strategy.

There were some limitations to be addressed in the future. First, the patients were from a single center and our study included a relatively small sample size. Although our study revealed several interesting findings, larger patient populations from multi-centers are needed for further validation and application of the prediction nomogram. Second, we excluded tumors less than 2.0 cm, since the parameter measurement might be unstable in small tumors. Third, the manual delineation of ITR and semi-automatic delineation of PTR might introduce some subjectivity. Finally, the follow-up time was too short to accurately evaluate the overall survival rate in this study.

In conclusion, quantitative DCE-MRI provides a promising tool for predicting MVI in HCC noninvasively and preoperatively. Additionally, the combined nomogram based on CR features and quantitative DCE parameters of ITR and PTR achieves excellent prediction performance. The predicted MVI risk classification can stratify the risk of recurrence after radical hepatectomy and aid in the selection of optimal surgical approaches.

### Supplementary information

ELECTRONIC SUPPLEMENTARY MATERIAL

## Data Availability

The datasets used and/or analyzed during the current study are available from the corresponding author upon reasonable request.

## Abbreviations

AFP: α-fetoprotein
AR: Anatomical resection
ART: Arterial fraction
BCLC: Barcelona Clinic Liver Cancer
*C*_DCE_: Combined DCE parameter
CR: Clinical-radiological
DCA: Decision curve analysis
DCE-MRI: Dynamic contrast-enhanced magnetic resonance imaging
DV: Distribution volume
*F*_a_: Arterial flow
*F*_p_: Portal-venous flow
*F*_t_: Total blood flow
HCC: Hepatocellular carcinoma
HMVI: High-risk of MVI
ICC: Intraclass correlation coefficient
ITR: Intra-tumoral region
LI-RADS: Liver Imaging Reporting and Data System
LMVI: Low-risk of MVI
MN: MVI negative
MP: MVI positive
MRI: Magnetic resonance imaging
MTT: Mean transit time
MVI: Microvascular invasion
NAR: Non-anatomical resection
PTR: Peritumoral region
RFS: Recurrence-free survival
ROC: Receiving operating curve
ROI: Region of interest
TTPVI: Two-trait predictor of venous invasion
