# Supplementary material for: Bi-regional dynamic contrast-enhanced MRI for prediction of microvascular invasion in solitary BCLC stage A hepatocellular carcinoma
Source: Insights Imaging. 2024 Jun 18;15:149. doi: 10.1186/s13244-024-01720-w (PMC11183021; doi:10.1186/s13244-024-01720-w)
Supplement: Supplementary file 1 — ELECTRONIC SUPPLEMENTARY MATERIAL [file 13244_2024_1720_MOESM1_ESM.pdf]

# Bi-regional dynamic contrast-enhanced MRI for prediction of microvascular invasion in solitary BCLC stage A hepatocellular carcinoma

## ELECTRONIC SUPPLEMENTARY MATERIAL

Insights Imaging (2024) Zhu Y, Feng B, Wang P, et al.

**Appendix E1** MR imaging acquisition protocol

The conventional liver MRI protocols at our institution includes the following sequence: (1) breath-hold axial fat-suppressed T1-weighted gradient recalled echo images including in-phase and opposed-phase acquired with three-dimensional (3D) liver acquisition with volume acceleration flexible (LAVA-Flex) sequences; (2) breath-hold coronal single-shot fast spin-echo T2-weighted imaging (T2WI); (3) respiratory-triggered axial PROPELLER T2-weighted imaging with fat suppression (T2WI/FS); and (4) respiratory-triggered axial diffusion weighted imaging (DWI) sequence included two $b$ values ($b$ values = 0 and 800 s/mm$^2$). Quantitative DCE-MRI was performed by using multiphase axial 3D spoiled-gradient recalled-echo sequences for liver acquisition with volume acceleration-extended volume (LAVA-XV) sequence with breath-hold. According to our previous study [1], pre-contrast T1 mapping with four different flip angles (3°, 6°, 9°, and 12°) was acquired before dynamic scanning for the determination of pre-contrast T1 values. Then a dynamic scan with 42 consecutive phases was performed, which shared the scanning parameters and range as T1 mapping, with a flip angle of 15° and temporal resolution of 4-6 s/phase. A bolus of gadodiamide (Omniscan 0.5 mmol/mL; GE Healthcare, Ireland) was injected using a standard dosage of 0.1 mmol/kg at a rate of 3.0 mL/s though an automated power injector, followed by flushing of 20 mL saline at the same rate. The intermittent breath holding method was adopted during DCE sequence scanning: every three consecutive phases were acquired in one breath hold with a break of 5–8 s for each cycle and repeated for 14 cycles. The total scanning time for DCE-MRI was 4–5 min. At last, a coronal LAVA-Flex sequence was acquired.

**References:**

1. Zhu Y, Zhou Y, Zhang W, Xue L, Li Y, Jiang J, et al (2021) Value of quantitative dynamic contrast-enhanced and diffusion-weighted magnetic resonance imaging in predicting extramural venous invasion in locally advanced gastric cancer and prognostic significance. Quant Imaging Med Surg 11(1):328-34

**Appendix E2** Perfusion DCE-MR image processing

The multi-phase DCE MR images were registered by GenIQ software package on Advantage Workstation version 4.7 (GE Healthcare, USA) for pre-processing prior to further analysis. The registered DCE-MR images were processed and analyzed using an inhouse program written on MATLAB R2018a (MathWorks, Natick, MA, USA). First, two round region of interest (ROI) was manually placed on abdominal aorta and main portal vein at level of porta hepatis for arterial input function (AIF) and portal vein input function (VIF) calculation, which were used as an estimation for the hepatic arterial and portal venous blood supply, respectively. Then, a third ROI was manually traced along the edge of the liver at the slice of maximum tumor diameter. The signal intensity on MRI was converted into an equivalent concentration of contrast material using multiple flip angles method and T1 fitting. Subsequently, the contrast concentration curves of the ROIs were calculated using a dual-input single compartment model, which depicts contrast material distribution after injection and predicts a change in the contrast concentration in the tissue as a function of time, C(t), using the below equation according to previously described [1, 2]:

$$\frac{dC(t)}{dt} = k_{1a} \cdot C_a\,(t) + k_{1p} \cdot C_p(t) - k_2 \cdot C(t)$$

where $C(t)$, $C_a(t)$, and $C_p(t)$ were the concentrations of contrast material in the tissue, aorta, and portal vein, and, respectively; $k_{1a}$, $k_{1p}$, and $k_2$ were constants for the aortic inflow rate, the portal venous inflow rate, and the outflow rate, respectively.

**References:**


1. Stocker D, Hectors S, Bane O, Vietti-Violi N, Said D, Kennedy P, et al (2021) Dynamic contrast-enhanced MRI perfusion quantification in hepatocellular carcinoma: comparison of gadoxetate disodium and gadobenate dimeglumine. Eur Radiol 31(12):9306-9315
2. Hectors SJ, Wagner M, Besa C, Bane O, Dyvorne HA, Fiel MI, et al (2016) Intravoxel incoherent motion diffusion-weighted imaging of hepatocellular carcinoma: Is there a correlation with flow and perfusion metrics obtained with dynamic contrast-enhanced MRI? J Magn Reson Imaging 44(4):856-864

**Table S1.** MR imaging acquisition protocol and main sequence parameters

| **Sequence** | **Plane** | **TR (ms)** | **TE (ms)** | **FOV (mm)** | **Matrix** | **FA (degrees)** | **ST/gap (mm)** | **NEX** | **Bandwidth (kHz)** | **Acquisition time** |
|---|---|---|---|---|---|---|---|---|---|---|
| LAVA-Flex T1WI | Axial | 5.1 | 1.4 | 400 | 288×224 | 12 | 5/0 | 1 | 166.67 | 14 (s) |
| SS-FSE T2WI | Coronal | 1816 | 68 | 400 | 288×288 | 90 | 4/1 | 1 | 83.33 | 45 (s) |
| FS T2WI Propeller | Axial | 8,000-10,000 | 96-100 | 400 | 320×320 | 90 | 5/1 | 2.5 | 50.0 | 3-4 (min) |
| SS-EPI DWI * | Axial | 8000-10,000 | 56-60 | 380 | 128×160 | 90 | 5/1 | 2 | 250 | 1.50 (min) |
| Precontrast LAVA-XV T1WI | Axial | 2.9 | 1.4 | 380 | 288×224 | 3, 6, 9, and 12 | 5/0 | 1 | 125 | 6 (s) |
| DCE-perfusion MRI | Axial | 2.9 | 1.4 | 380 | 288×224 | 15 | 5/0 | 1 | 125 | 4-6 (min) |
| Postcontrast LAVA- | Coronal | 4.2 | 1.9 | 400×36 | 352×25 | 15 | 4/0 | 1 | 200 | 16 (s) |

| | | |
|---|---|---|
| Flex T1WI | 0 | 6 |

[*] Acquired using respiratory trigger technique and 2 *b*-values were used (0 and 800 s/mm$^2$)

*DCE*, dynamic-contrast enhanced; *DWI*, diffusion-weighted imaging; *FA*, Flip angle; *FOV*, Field of view; *FS*, fat suppression; *LAVA-Flex*, liver acquisition with volume acceleration; *LAVA-XV*, liver acquisition with volume acceleration-extended volume; *NEX*, number of excitations; *SS-EPI*, Single-shot echo-planar imaging; *SS-FSE*, single-shot fast-recovery fast spin-echo; *ST*, slice thickness; *T1WI*, T1-weighted imaging; *T2WI*, T2-weighted imaging; *TE*, echo time; *TR*, repetition time

**Table S2.** Definition of each MR imaging feature in this study

| MR imaging feature | Definitions |
|---|---|
| **LI-RADS major features** [1] | |
| Tumor size, cm | Largest outer-edge-to-outer-edge dimension of an observation. |
| No-rim arterial phase hyperenhancement | Nonrim-like enhancement in arterial phase unequivocally greater in whole or in part than liver. |
| Non-peripheral washout | Nonperipheral visually assessed temporal reduction in enhancement in whole or in part relative to composite liver tissue from earlier to later phase resulting in hypoenhancement in the extracellular phase. |
| Enhancing capsule | Smooth, uniform, sharp border around most or all of an observation, unequivocally thicker or more conspicuous than fibrotic tissue around background nodules, and visible as enhancing rim in PVP, DP, or TP. |
| **LI-RADS ancillary features (favoring HCC in particular)** | |
| Non-enhancing capsule | Capsule appearance not visible as an enhancing rim. |
| Nodule-in-nodule architecture | Presence of smaller inner nodule within and having different imaging features than larger outer nodule. |
| Mosaic architecture | Presence of randomly distributed internal nodules or compartments, usually with different imaging features. |
| Fat in mass, more than adjacent liver | Relative paucity of fat in solid mass compared to steatotic liver OR in inner nodule relative to steatotic outer nodule. |
| Blood products in mass | Intralesional hemorrhage in absence of biopsy, trauma, or intervention. Perilesional hemorrhage |

| | |
|---|---|
| | may or may not be present. |
| **LI-RADS ancillary features (favoring malignancy, not HCC in particular)** | |
| Restricted diffusion | Intensity on DWI, not attributable solely to T2 shine-through, unequivocally higher than liver and/or ADC unequivocally lower than liver |
| Mild-moderate T2 hyperintensity | Signal intensity on T2-weighted images mildly or moderately higher than liver and similar to or less than non-iron-overloaded spleen |
| Corona enhancement | Periobservational enhancement in late arterial phase or early PVP attributable to venous drainage from tumor. |
| Fat sparing in solid mass | Relative paucity of fat in solid mass compared to steatotic liver OR in inner nodule relative to steatotic outer nodule |
| Iron sparing in solid mass | Paucity of iron in solid mass relative to iron-overloaded liver or in inner nodule relative to outer |
| | siderotic nodule. |
| **Non-LIRADS imaging features** | |
| Cirrhosis | Imaging manifestations of cirrhosis including morphological alteration (global atrophy, segmental volume redistribution, and regional or focal parenchymal contraction), parenchymal alteration (parenchymal nodules, fibrotic scars surrounding parenchymal nodules, confluent hepatic fibrosis, nonspecific parenchymal heterogeneity, fat, and iron accumulation), vascular alterations (hepatic artery, portal vein, and intrahepatic veins, etc.), biliary alterations (peribiliarycysts), functional alterations in HBP (altered expression levels of hepatocyte transporters), musculoskeletal manifestations (sarcopenia), and portal hypertension (portal-systemic collaterals, spleen, fluid retention, submucosal edema) [1]. |

| | |
|---|---|
| Tumor margin | Defined on the portal venous phase and/or delayed phase, and was categorized as i) smooth margin presenting as nodular tumors with smooth contour, and ii) non-smooth margin presenting as an irregular margin that had budding portion at the tumor periphery protruding into the liver parenchyma [2]. |
| Tumor capsule | A thin, linear and hyperenhancement structure surrounded the tumor border in the portal venous and/or delayed phase and grouped into absent, incomplete, and intact types [3]. |
| TTPVI | The presence of internal arteries visible in the arterial phase and the absence of hypointense halo in a post-arterial phase [4]. |

Note—Unless otherwise indicated, all MR imaging features were defined according to the LI-RADS version 2018 [1]

*ADC*, apparent diffusion coefficient; *DP*, delayed phase; *DWI*, diffusion-weighted imaging; *HBP*, hepatobiliary phase; *HCC*, hepatocellular carcinoma; *LI-RADS*, Liver Imaging Reporting and Data System; *PVP*, portal venous phase; *TP*, transitional phase; *TTPVI*, two-trait predictor of venous invasion

**References:**


1. American College of Radiology website. CT/MRI LI-RADS v2018. www.acr.org/Clinical-Resources/Reporting-and-Data-Systems/LI-RADS/CT-MRI-LI-RADSv2018.Accessed January 2021
2. Dong SY, Wang WT, Chen XS, Yang YT, Zhu S, Zeng MS, et al. Microvascular invasion of small hepatocellular carcinoma can be preoperatively predicted by the 3D quantification of MRI. Eur Radiol 2022;32(6):4198-4209.
3. Liu B, Zeng Q, Huang J, Zhang J, Zheng Z, Liao Y, et al. IVIM using convolutional neural networks predicts microvascular invasion in HCC. Eur Radiol 2022;32(10):7185-7195.
4. Renzulli M, Brocchi S, Cucchetti A, Mazzotti F, Mosconi C, Sportoletti C, et al. Can Current Preoperative Imaging Be Used to Detect Microvascular Invasion of Hepatocellular Carcinoma? Radiology 2016;279(2):432-442.

**Table S3.** Interobserver variability for DCE perfusion quantitative parameters measurement of primitive lesions in patients with hepatocellular carcinoma

| DCE perfusion parameters | ICC | 95% Confidence Interval | *P* value |
|---|---|---|---|
| **Intra-tumoral region** | | | |
| ART (%) | 0.932 | 0.883 – 0.982 | < 0.001 |
| Fa (ml/min/100 g) | 0.886 | 0.824 – 0.944 | < 0.001 |
| Fp (ml/min/100 g) | 0.905 | 0.863 – 0.970 | < 0.001 |
| Ft (ml/min/100 g) | 0.887 | 0.842 – 0.933 | < 0.001 |
| DV (%) | 0.953 | 0.922 – 0.985 | < 0.001 |
| MTT (sec) | 0.894 | 0.832 – 0.942 | < 0.001 |
| **Peritumoral region** | | | |
| ART (%) | 0.874 | 0.835 – 0.918 | < 0.001 |
| Fa (ml/min/100 g) | 0.825 | 0.783 – 0.877 | < 0.001 |
| Fp (ml/min/100 g) | 0.804 | 0.753 – 0.860 | < 0.001 |
| Ft (ml/min/100 g) | 0.893 | 0.844 – 0.951 | < 0.001 |
| DV (%) | 0.864 | 0.803 – 0.924 | < 0.001 |
| MTT (sec) | 0.858 | 0.821 – 0.889 | < 0.001 |

*ART*, arterial fraction; *DCE*, dynamic-contrast enhanced; *DV*, distribution volume; $F_a$, arterial blood flow; $F_p$, portal venous blood flow; $F_t$, total blood flow; *ICC*, intraclass correlation coefficient; *MTT*, mean transit time

**Table S4.** Inter-observer agreement for radiological characteristics

| Features | Reader 1 | Reader 2 | Disagreement | Kappa (95 % CI) * |
|---|---|---|---|---|
| **LI-RADS major features** | | | | |
| Tumor size, cm | | | 0 | 1.000 (1.000, 1.000) |
| < 2.0 | 0 | 0 | | |
| ≥ 2.0 | 133 | 133 | | |
| No-rim arterial phase hyperenhancement | | | 7 | 0.734 (0.607, 0.861) |
| Absent | 7 | 9 | | |
| Present | 126 | 124 | | |
| Non-peripheral washout | | | 6 | 0.864 (0.758, 0.970) |
| Absent | 27 | 29 | | |
| Present | 106 | 104 | | |
| Enhancing capsule | | | 12 | 0809 (0.705, 0.913) |
| Absent | 51 | 49 | | |
| Present | 83 | 85 | | |
| **LI-RADS ancillary features (favoring HCC in particular)** | | | | |
| Non-enhancing capsule | | | 7 | 0.770 (0.607, 0.933) |
| Absent | 116 | 115 | | |
| Present | 17 | 18 | | |
| Nodule-in-nodule architecture | | | 6 | 0.674 (0.431, 0.917) |
| Absent | 114 | 112 | | |
| Present | 9 | 11 | | |
| Mosaic architecture | | | 13 | 0.794 (0.688, 0.900) |
| Absent | 83 | 80 | | |
| Present | 50 | 53 | | |
| Fat in mass, more than adjacent liver | | | 16 | 0.695 (0.556, 0.834) |
| Absent | 98 | 96 | | |
| Present | 35 | 37 | | |
| Blood products in mass | | | 15 | 0.701 (0.562, 0.840) |
| Absent | 102 | 97 | | |
| Present | 31 | 36 | | |
| **LI-RADS ancillary features (favoring malignancy, not HCC in particular)** | | | | |
| Restricted diffusion | | | 0 | 1.000 (1.000, 1.000) |
| Absent | 0 | 0 | | |
| Present | 133 | 133 | | |

| | | | | | |
|---|---|---|---|---|---|
| Mild-moderate T2 hyperintensity | | | 0 | 1.000 1.000) | (1.000, |
| Absent | 0 | 0 | | | |
| Present | 133 | 133 | | | |
| Corona enhancement | | | 11 | 0.807 0.915) | (0.699, |
| Absent | 91 | 92 | | | |
| Present | 42 | 41 | | | |
| Fat sparing in solid mass | | | 5 | 0.762 0.962) | (0.562, |
| Absent | 121 | 122 | | | |
| Present | 12 | 11 | | | |
| Iron sparing in solid mass | | | 3 | 0.716 1.000) | (0.410, |
| Absent | 128 | 127 | | | |
| Present | 5 | 6 | | | |
| **Non-LIRADS imaging features** | | | | | |
| Cirrhosis | | | 11 | 0.834 0.928) | (0.740, |
| Absent | 70 | 73 | | | |
| Present | 63 | 60 | | | |
| Tumor margin | | | 12 | 0.806 0.910) | (0.702, |
| Smooth | 83 | 85 | | | |
| Non-smooth | 50 | 48 | | | |
| Tumor capsule | | | 11 | 0.825 0.923) | (0.727, |
| Complete | 80 | 85 | | | |
| Incomplete/absent | 53 | 48 | | | |
| TTPVI | | | 7 | 0.874 0.964) | (0.784, |
| Absent | 92 | 95 | | | |
| Present | 41 | 38 | | | |

Except where indicated otherwise, data are expressed as numbers with percentages in parentheses

* Data are expressed as kappa coefficient with 95% CIs in parentheses

*CI*, confidence interval; *HCC*, hepatocellular carcinoma; *LI-RADS*, Liver Imaging Reporting and Data System; *TTPVI*, two-trait predictor of venous invasion

**Table S5.** Construction of clinic-radiological, DCE, and combined models through multivariate logistic analysis

| Models * | β | S.E. | Wald | *P* value | OR | 95% CI for OR | |
|---|---|---|---|---|---|---|---|
| | | | | | | Lower | Upper |
| *Clinic-radiological model* | | | | | | | |
| AFP, ng/ml | | | | | | | |
| ≤ 20 vs. 20~400 | 1.622 | 0.669 | 5.880 | 0.015 | 5.064 | 1.365 | 18.788 |
| ≤ 20 vs. > 400 | 2.675 | 0.781 | 11.722 | < 0.001 | 14.517 | 3.139 | 67.148 |
| Corona enhancement (Present) | 1.471 | 0.557 | 6.969 | 0.008 | 4.355 | 1.461 | 12.983 |
| TTPVI (Present) | 1.685 | 0.605 | 7.767 | 0.005 | 5.395 | 1.649 | 17.652 |
| Constant | | | | | | | |
| *Intra-tumoral model* | | | | | | | |
| $F_t$ (ml/min/100 g) | 0.048 | 0.010 | 25.343 | <0.001 | 1.049 | 1.030 | 1.069 |
| MTT (sec) | -0.167 | 0.075 | 4.972 | 0.026 | 0.846 | 0.731 | 0.980 |
| Constant | -2.123 | 1.098 | 3.741 | 0.053 | | | |
| *Peritumoral model* | | | | | | | |
| ART (%) | 0.171 | 0.044 | 12.927 | < 0.001 | 1.186 | 1.088 | 1.293 |

| | | | | | | | |
|---|---|---|---|---|---|---|---|
| Constant | -5.407 | 1.267 | 18.222 | < 0.001 | 0.004 | | |
| *DCE model* | | | | | | | |
| $F_t$-T (mL/min/100 g) | 0.026 | 0.009 | 7.950 | 0.005 | 1.026 | 1.008 | 1.004 |
| ART-P (%) | 0.170 | 0.048 | 12.761 | < 0.001 | 1.185 | 1.080 | 1.301 |
| Constant | -10.052 | 2.308 | 18.966 | < 0.001 | 0.000 | | |
| *Combined model* | | | | | | | |
| AFP, ng/mL | | | | | | | |
| ≤ 20 vs. 20~400 | 2.346 | 1.028 | 5.206 | 0.023 | 10.446 | 1.392 | 78.386 |
| ≤ 20 vs. > 400 | 4.630 | 1.352 | 11.726 | < 0.001 | 102.554 | 7.244 | 1451.885 |
| Corona enhancement (Present) | 2.394 | 0.916 | 6.825 | 0.009 | 10.957 | 1.818 | 66.021 |
| TTPVI (Present) | 3.291 | 1.213 | 7.369 | 0.007 | 26.880 | 2.497 | 289.410 |
| $C_{DCE}$ | 1.315 | 0.343 | 14.683 | < 0.001 | 3.726 | 1.901 | 7.300 |
| Constant | -4.079 | 1.116 | 13.356 | < 0.001 | 0.017 | | |

* A stepwise forward method was used to assess the best independent predictor of microvascular invasion

*AFP*, alpha fetoprotein; *ART*, arterial fraction; *β*, coefficient; $C_{DCE}$, combined quantitative parameter of DCE-MRI; *CI*, confidence interval; *DCE*, dynamic-contrast enhanced; $F_t$, total blood flow; *MTT*, mean transit time; *OR*, odds ratio; *P*, peritumoral region; *S.E.*, standard error; *T*, intra-tumoral region; *TTPVI*, two-trait predictor of venous invasion

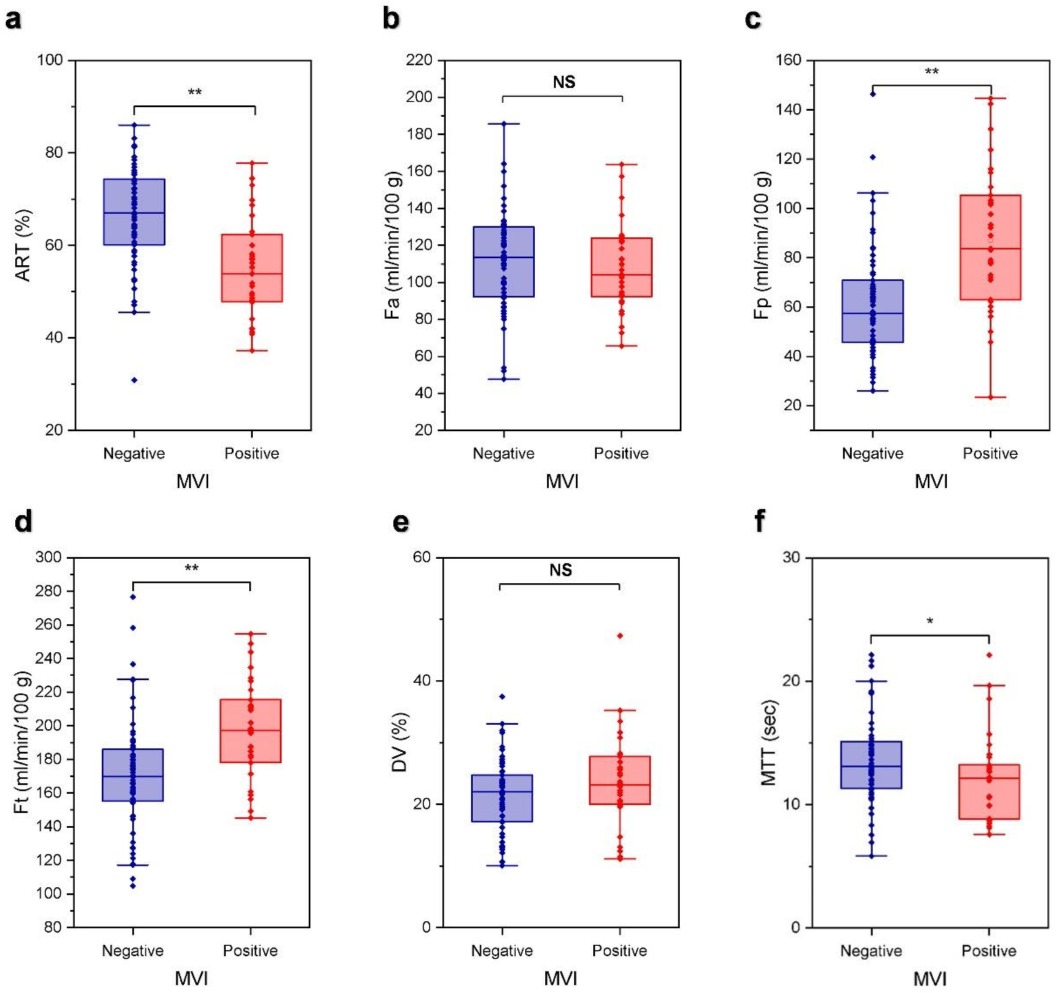


**Fig. S1.** Box and whisker plots of the show the distribution of each perfusion DCE parameter of intra-tumoral region in the training set between MVI positive and MVI negative groups. Box and whisker plots of ART (**a**), $F_a$ (**b**), $F_p$ (**c**), $F_t$ (**d**), DV (**e**) and MTT (**f**) between MVI positive and MVI negative groups, respectively. Boxes show the upper and lower quartiles, and horizontal lines within boxes indicate median values, whiskers indicate first quartile minus 1.5 times inner quartile range and third quartile plus 1.5 times inner quartile range, and circles indicate outliers. Asterisks indicate significant differences between MVI positive and MVI negative groups ($P < 0.05$). NS indicate no significant differences between MVI positive and MVI negative groups ($P > 0.05$). *MVI*, microvascular invasion.

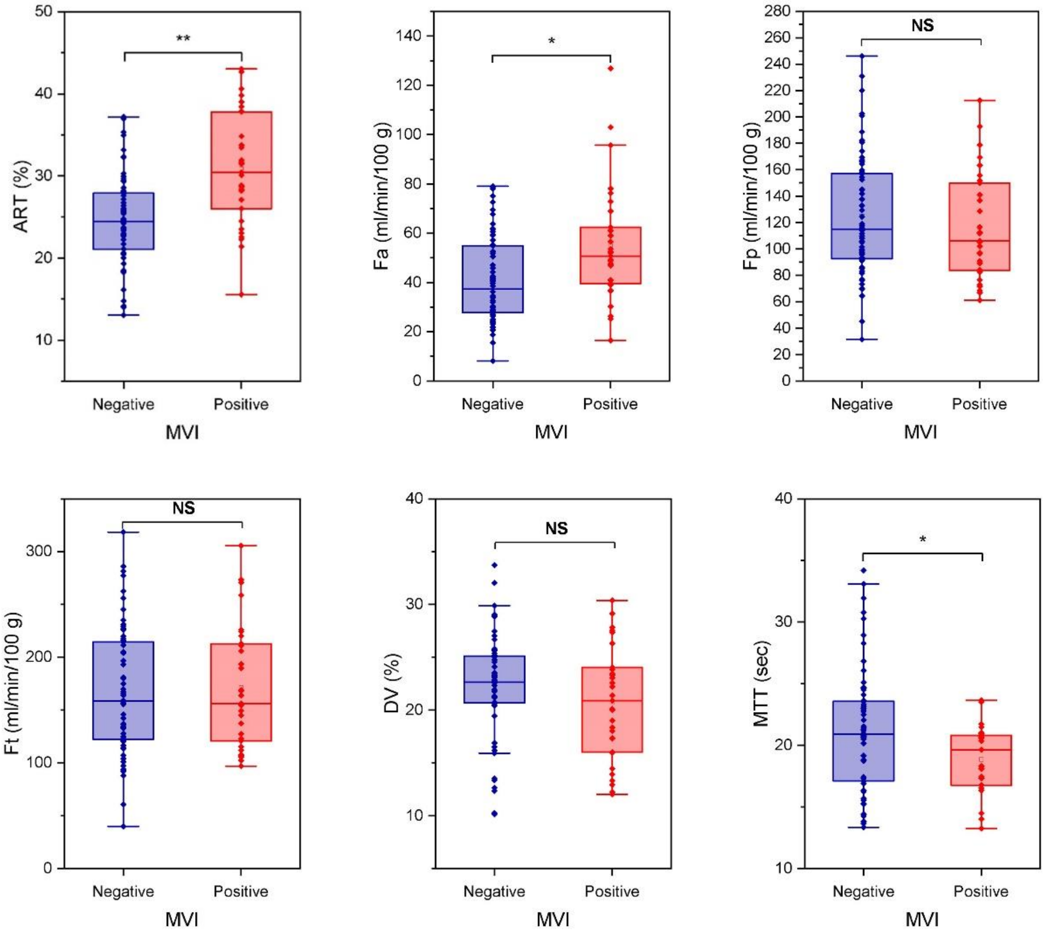


**Fig. S2.** Box and whisker plots of the show the distribution of each perfusion DCE parameter of peri-tumoral region in the training set between MVI positive and MVI negative groups. Box and whisker plots of ART (**a**), $F_a$ (**b**), $F_p$ (**c**), $F_t$ (**d**), DV (**e**) and MTT (**f**) between MVI positive and MVI negative groups, respectively. Boxes show the upper and lower quartiles, and horizontal lines within boxes indicate median values, whiskers indicate first quartile minus 1.5 times inner quartile range and third quartile plus 1.5 times inner quartile range, and circles indicate outliers. Asterisks indicate significant differences between MVI positive and MVI negative groups ($P$ < 0.05). NS indicate no significant differences between MVI positive and MVI negative groups ($P$ > 0.05). *MVI*, microvascular invasion.

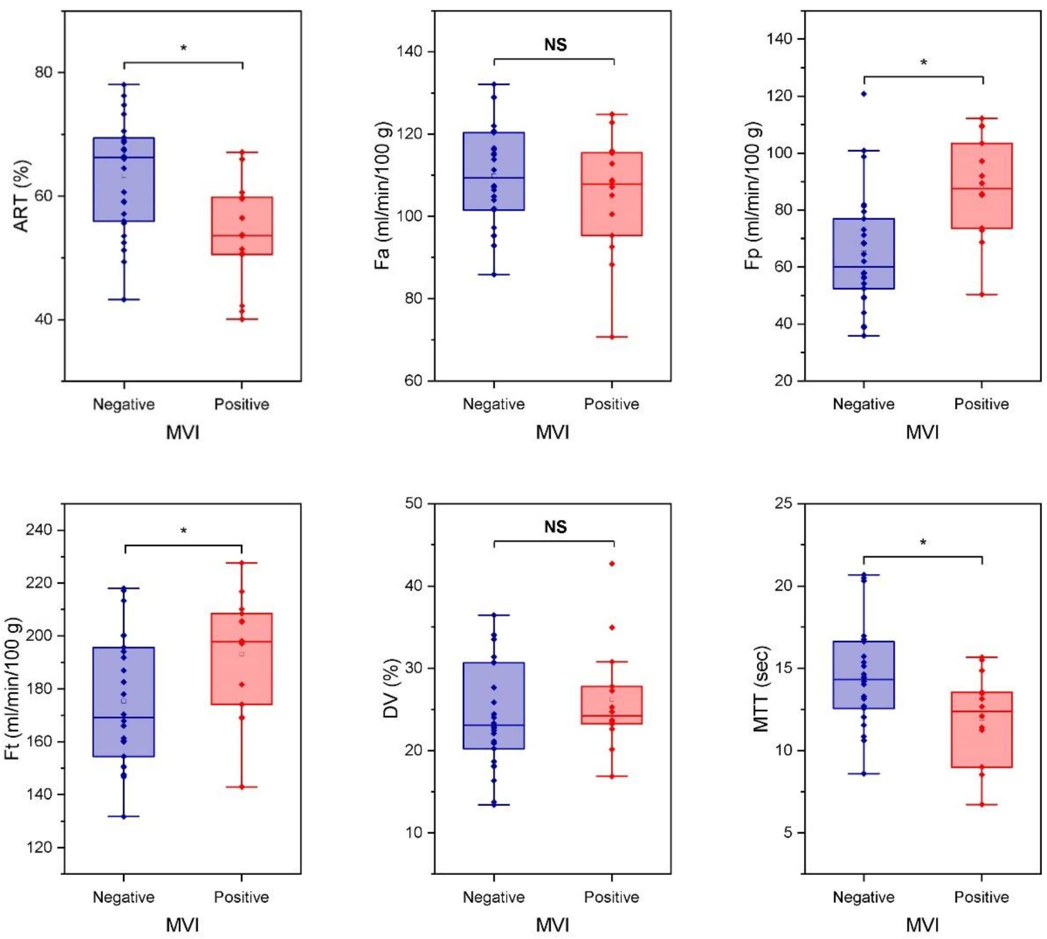


**Fig. S3.** Box and whisker plots of the show the distribution of each perfusion DCE parameter of intra-tumoral region in the validation set between MVI positive and MVI negative groups. Box and whisker plots of ART (**a**), $F_a$ (**b**), $F_p$ (**c**), $F_t$ (**d**), DV (**e**) and MTT (**f**) between MVI positive and MVI negative groups, respectively. Boxes show the upper and lower quartiles, and horizontal lines within boxes indicate median values, whiskers indicate first quartile minus 1.5 times inner quartile range and third quartile plus 1.5 times inner quartile range, and circles indicate outliers. Asterisks indicate significant differences between MVI positive and MVI negative groups ($P < 0.05$). NS indicate no significant differences between MVI positive and MVI negative groups ($P > 0.05$). *MVI*, microvascular invasion.

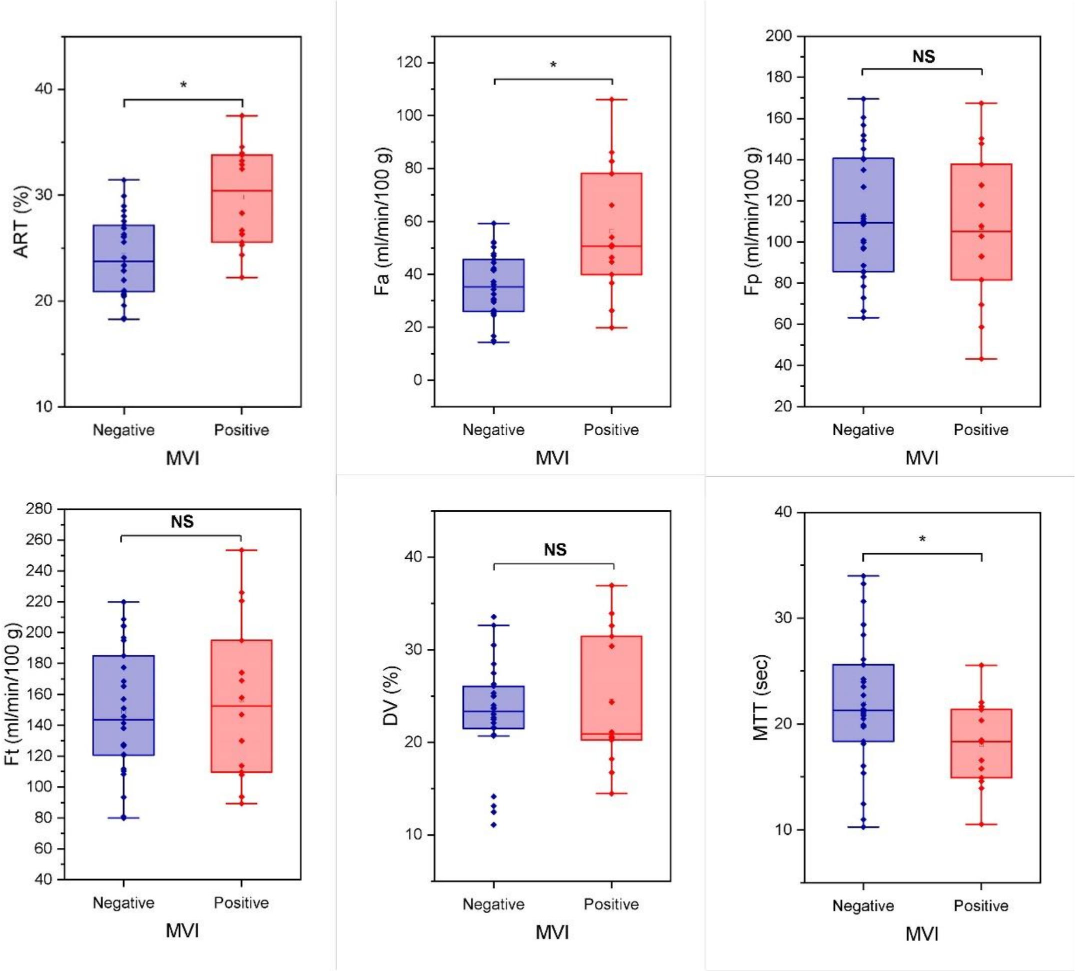


**Fig. S4.** Box and whisker plots of the show the distribution of each perfusion DCE parameter of peri-tumoral region in the validation set between MVI positive and MVI negative groups. Box and whisker plots of ART (**a**), $F_a$ (**b**), $F_p$ (**c**), $F_t$ (**d**), DV (**e**) and MTT (**f**) between MVI positive and MVI negative groups, respectively. Boxes show the upper and lower quartiles, and horizontal lines within boxes indicate median values, whiskers indicate first quartile minus 1.5 times inner quartile range and third quartile plus 1.5 times inner quartile range, and circles indicate outliers. Asterisks indicate significant differences between MVI positive and MVI negative groups ($P < 0.05$). NS indicate no significant differences between MVI positive and MVI negative groups ($P > 0.05$). *MVI*, microvascular invasion.

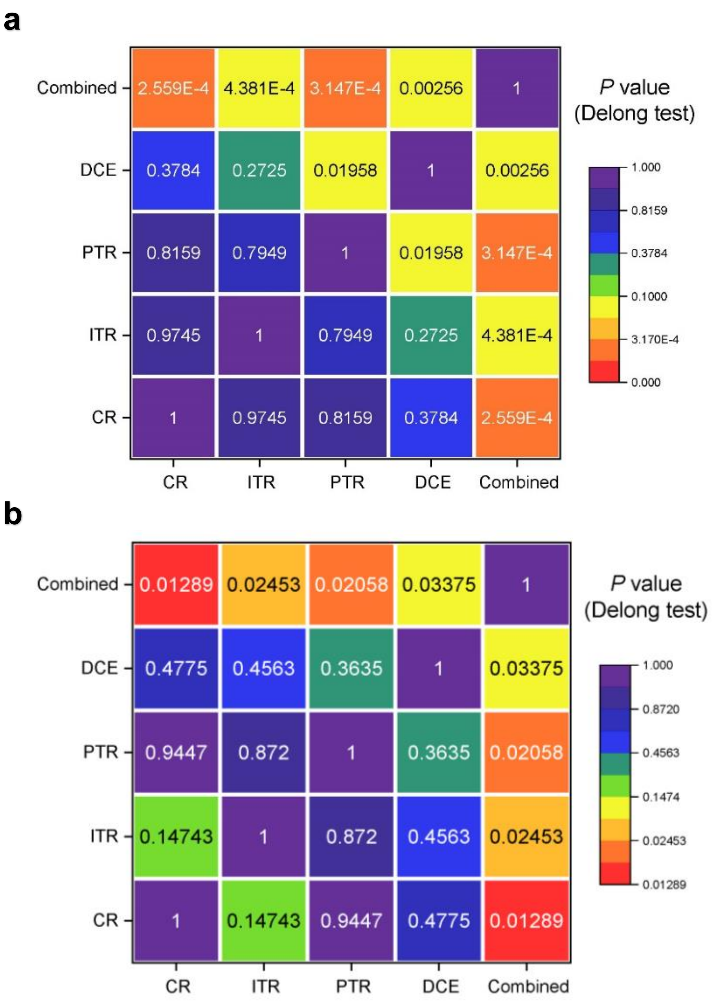


**Fig. S5.** Heat maps show the *p* values of the Delong test used for comparing the areas under the curves of different predicting models for microvascular invasion in hepatocellular carcinoma. *CR*, clinic-radiological; *DCE*, dynamic-contrast enhanced; *ITR*, intra-tumoral region; *PTR*, peritumoral region
